# Ruminants reveal Eocene Asiatic palaeobiogeographical provinces as the origin of diachronous mammalian Oligocene dispersals into Europe

**DOI:** 10.1038/s41598-021-96221-x

**Published:** 2021-09-06

**Authors:** Bastien Mennecart, Manuela Aiglstorfer, Yikun Li, Chunxiao Li, ShiQi Wang

**Affiliations:** 1 Naturhistorisches Museum Wien, Burgring 7, 1010 Vienna, Austria; 2 Naturhistorisches Museum Basel, Augustinergasse 2, 4001 Basel, Switzerland; 3 Naturhistorisches Museum Mainz/Landessammlung Für Naturkunde Rheinland-Pfalz, Reichklarastraße 10, 55116 Mainz, Germany; 4 Center for Research and Education On Biological Evolution and Environment, Nanjing Univeristy, Nanjing, 210023 China; 5 Key Laboratory of Vertebrate Evolution and Human Origins of Chinese Academy of Sciences, Institute of Vertebrate Paleontology and Paleoanthropology, Chinese Academy of Sciences, Beijing, 100044 China; 6 CAS Center for Excellence in Life and Paleoenvironment, Beijing, 100044 China

**Keywords:** Palaeontology, Palaeontology, Phylogenetics, Biogeography

## Abstract

Faunal provincialism between the North and South parts of Eastern Asia is shown to have been in place since the late Eocene. This provincialism structured the mammalian dispersals across Eurasia for millions of years and provides insights into both palaeonvironments and palaeoclimate zonation. In addition, this study reveals the oldest record of a crown ruminant (*Iberomeryx* from Shinao, China). Ecologically, as well as economically, ruminant artiodactyls are one of the most important large mammal groups today. The revision of the ruminants from the Shinao Formation, from the Caijiachong marls and Xiaerhete, resulted in two new taxa and shows that the different provinces were populated by distinct taxa living in different environments, dominated by the monsoon in the South and drier conditions in the North. Evaluating this result in a Eurasian context demonstrates that the dispersals from Asia to Europe was complex. These results confirm that there were at least two dispersal events, distinct in space and time: the Grande-Coupure from Northern and Central Asia along the North ca. 34 Mya and the *Bachitherium* dispersal event from the Southern province along a southerly route ca. 31 Mya.

## Introduction

The fossil record of Asia holds most of the spectacular findings in vertebrate palaeontology of the several last decades and many questions in mammal evolution can be tackled now thanks to these discoveries. The Ruminantia (crown group comprising the Pecora: pronghorns, giraffes, deer, cows, and musk deer; the Tragulidae: mouse deer; and all fossil representatives until their last shared ancestor) is an ecologically and economically important group of artiodactyls. Like for other mammals, the origin of that clade is hidden as well somewhere in the long Asian geological history. Most likely their roots go back to the late middle Eocene, at ca. 45 Mya^1,2^. Traditionally, the Eocene was considered as rather homogenous in climate and faunal composition at a large scale in Asia^3^. However, while the South-eastern part of Asia has shown very few changes in a warm and humid climate and environment since the Eocene^4^, Northern Asia faced a progressive but complex transition from warm and humid subtropical environments during the Eocene to steppe environments in the Pliocene, e.g.^3–5^. The aridification in Northern and Central Asia (recorded from the early Bartonian, ca. 40–37 Mya, onwards in Central Asia^6–9^) was triggered by a variety of factors, e.g.^9–12^, which led to a strong latitudinal environmental and climatic zonation during the middle and the late Eocene, e.g.^6,13^. Despite this environmental heterogeneity, a relative homogenous fauna is described in Asia during that time.

All of the earliest ruminants are grouped into the Asian family Archaeomerycidae *sensu*^2^ (including *Archaeomeryx*, *Xinjiangmeryx*, *Indomeryx*, *Miomeryx*, and *Notomeryx*) that did not survive the Eocene/Oligocene transition^2^. Lophiomeryicidae are another clade of stem ruminants. The oldest lophiomerycid appeared during the late middle Eocene (*Zhailimeryx hetii*^14^) and did not diversify until the late Eocene and Oligocene when the family was distributed across the whole of Eurasia before it disappeared during the latest Oligocene^14–16^. Besides the co-occurence of Archaeomerycidae and Lophimomerycidae, the late Eocene also shows the first large scale diversification of ruminants with the appearance of Tragulidae in Southeast Asia^17^, “Gelocidae” and Praetragulidae (*sensu*^2^) in Central Asia and Bachitheriidae in the Southeast Europe^18,19^. The Tragulidae are the sole still surviving ruminant from all the above-mentioned families known during the Eocene.

Key localities for a better understanding of the Asian terrestrial ecosystem modifications during the Paleogene are the Shinao Formation and the Caijiachong (= Tsaichiachung) marls, as well as the Xiaerhete locality, where a diversified fauna has been discovered. The Shinao Formation (Southwestern Guizhou and adjoining Eastern Yunnan, China) yielded a relatively diverse and unique large mammal fauna includig the genus *Lophiomeryx*^20^. For a long time the Formation was dated to the early Oligocene, as *Lophiomeryx* was considered diagnostic for a post-Eocene age. New and revised data on the stratigraphic range of Lophiomerycidae (including the reappraisals of other Asian localities) and the faunal content of the Shinao Formation now proves a late Eocene age for the locality^2,14,20–25^.

Like the Shinao Formation, the Caijiachong marls (Qujing Yunnan, China) have long been considered as early Oligocene of age^26^. However, considering the great similarity of this fauna with the one of the Lunan Formation and of Ardyn Obo^26,27^, we agree with Vislobokova^21^ that the Caijiachong fauna should be assigned to the late Eocene, as well as Xiaerhete based on preliminary analyses^28^.

Here we reassess the late Eocene ruminants from the Shinao Formation and the specimens attributed to cf. *Miomeryx* sp. from the Caijiachong marls and we describe a new ruminant specimen from the late Eocene from Xiaerhete. The re-description of these specimens leads to the erection of a new genus and two new species and reveals the by far oldest crown ruminant, the tragulid *Iberomeryx*. This study shows for the first time that two latitudinal palaeoenvironmental provinces shaped the Eurasian dispersal patterns for more than 10 million years and were already present during the Eocene. It aids in a better understanding of Cenozoic Eurasian palaeobiogeography and the structure of palaeoclimate zonation, as well as the spreading of mammals across the continent during their evolutionary history. Indeed, the Northern and Central Asiatic *Lophiomeryx*, living in more arid environments, is known in Western Europe from the Grande-Coupure dispersal event (34 Mya), while the Southern Asiatic *Iberomeryx*, living in more humid environments, is known in Western Europe from the *Bachitherium* dispersal event (31 Mya).

## Systematic palaeontology

Mammalia Linnaeus, 1758^29^.

Artiodactyla Owen, 1848^30^.

Ruminantia Scopoli, 1777^31^.

Infraorder Tragulina Flower, 1883^32^.

Family Lophiomerycidae Janis, 1987^33^.

### Included genera

*Lophiomeryx*, *Zhailimeryx*, *Krabimeryx*, *Chiyoumeryx* nov. gen.

Genus *Krabimeryx* Métais, Chaimanee, Jaeger, and Ducroq, 2001^17^.

### Etymology

*Krabi—*from Krabi Basin, where the fossils were found, and—*meryx* is the Greek word for ruminant.

### Diagnosis [modified after Métais et al.^17^]

Small primitive ruminant with lower molars morphologically close to those of *Zhailimeryx*. *Krabimeryx* differs from *Zhailimeryx* in: more laterally compressed lingual cuspids in the lower molars; an entoconid displaced to anterior with respect to the hypoconid; the lack of both a paraconid and a hypoconulid in m1 and m2; a p4 with a mesolingual conid that is located more posterior and less individualized; a p4 without a distinct posterolingual conid. *Krabimeryx* differs from *Lophiomeryx* by less selenodont labial cuspids in the lower molars, the presence of a developed external postmetacristid, and by a distinct groove on the anterior side of the entoconid, the entoconidian groove. *Krabimeryx* can be distinguished from *Iberomeryx* in having a well-marked entoconidian groove; the lack of a clear external postprotocristid; the third lobe of m3 not forming a complete buckle; and a more transversely compressed hypoconulid in the m3. *Krabimeryx* possesses a huge notch in lingual view between the entoconid and the third lobe in the m3.

### Type species

*Krabimeryx primitivus* Métais, Chaimanne, Jaeger, and Ducroq, 2001^17^.

### Included species

*Krabimeryx gracilis* nov. comb. (Miao, 1982^20^).

*Krabimeryx gracilis* nov. comb. (Miao, 1982^20^).

**Figure 1:**
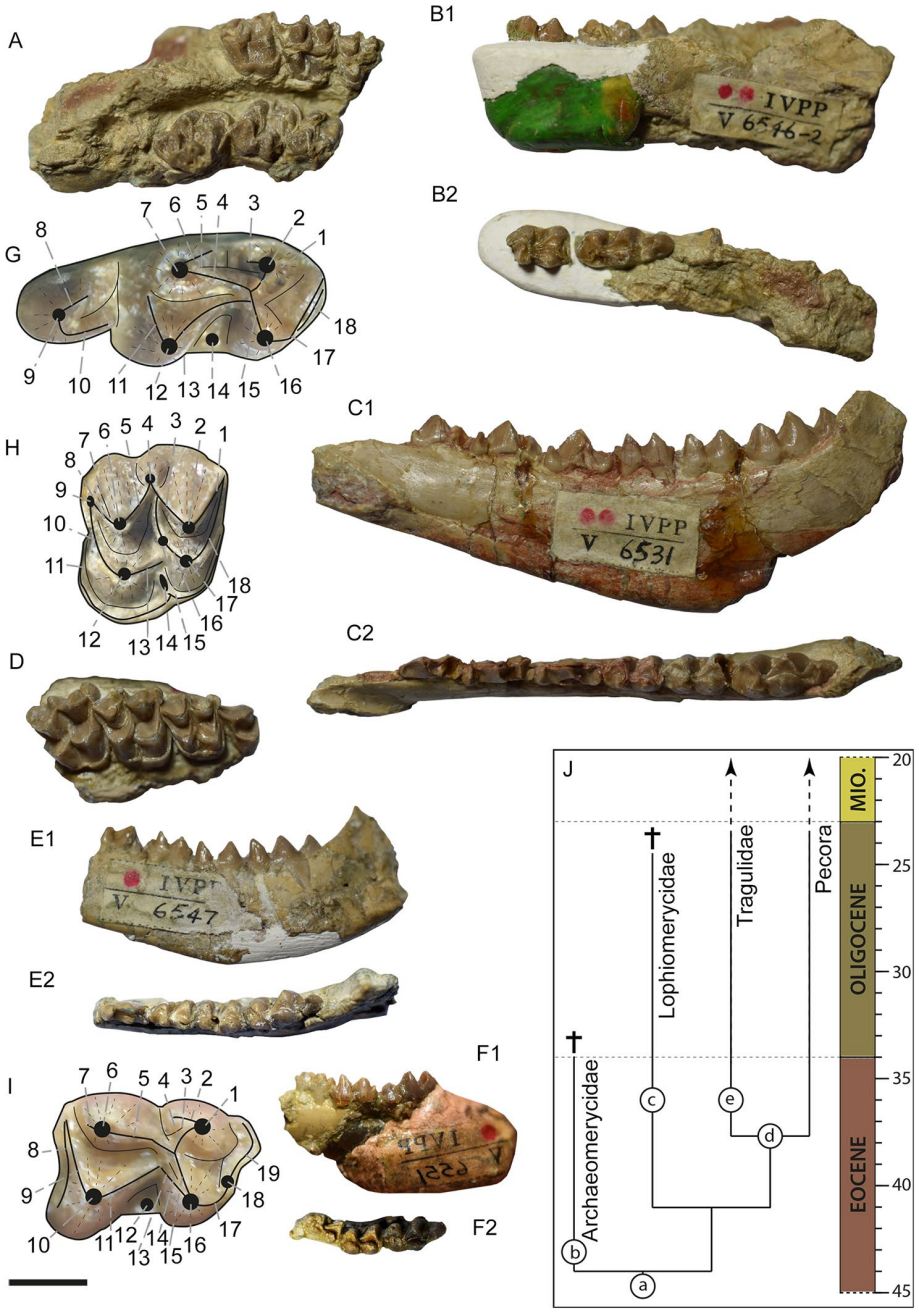
Dentition of *Krabimeryx gracilis* nov. comb. (Miao, 1982)^20^ (**A**, **B**, **G**, **H**), *Chiyoumeryx* nov. gen. *shinaoensis* (Miao, 1982)^20^ (**C**, **D**), *Chiyoumeryx* nov. gen. *flavimperatoris* nov. sp. (**E**) and *Iberomeryx miaoi* nov. sp. (**F**–**I**). *Krabimeryx gracilis* nov. comb. (Miao, 1982)^20^: (**A**) IVPP V 6546-1 (holotype), partial skull with right and left M1–M3; (**B**) IVPP V 6546-2 (holotype), right fragmented mandible with m2–m3. *Chiyoumeryx* nov. gen. *shinaoensis* (Miao, 1982)^20^: (**C**) IVPP V 6531 (holotype), right mandible with p2–m3 and tooth socket of p1; (**D**) IVPP V 6532 (paratype), right fragmented maxillary with P4-M3. *Chiyoumeryx* nov. gen. *flavimperatoris* nov. sp.: (**E**) IVPP V 6547 (holotype), right mandible with p4–m3; *Iberomeryx miaoi* nov. sp.: (**F**) IVPP V 6551 (holotype), left mandible with m1–m3 (mirrored); (**G**) lower molar Lophiomerycidae dental nomenclature (based on the m3 of IVPP 6546-2): 1 internal postmetacristid, 2 metaconid, 3 external postmetacristid, 4 internal preentocristid, 5 entoconidian groove, 6 external preentocristid, 7 entoconid, 8 posthypoconulidcristid, 9 hypoconulid, 10 prehypoconuldicristid, 11 posthypocristid, 12 hypoconid, 13 prehypocristid, 14 ectostylid, 15 postprotocristid, 16 protoconid, 17 preprotocristid, 18 anterior cingulid; (**H**) upper molar Lophiomerycidae dental nomenclature (based on the M2 of IVPP 6546-1): 1 postmetacrista, 2 metacone, 3 premetacrista, 4 mesostyle, 5 postparacrista, 6 paracone, 7 paraconid labial groove, 8 preparacrista, 9 parastyle, 10 preprotocrista, 11 anterolingual cingulum, 12 protocone, 13 postprotocrista, 14 entostyle, 15 additional cone, 16 premetaconulecrista, 17 metaconule, 18 postmetaconulecrista; (**I**) lower molar Tragulidae dental nomenclature (based on the m2 of IVPP V 6551, reversed): 1 metaconid, 2 external postmetacristid, 3 *Dorcatherium* fold, 4 internal postmetacristid, 5 preentocristid, 6 entoconid, 7 postentocristid, 8 posterior cingulid, 9 posthypocristid, 10 hypoconid, 11 prehypocristid, 12 ectostylid, 13 external postprotocristid, 14 *Tragulus* fold, 15 internal postprotocristid, 16 protoconid, 17 preprotocristid, 18 paraconid, 19 preparacristid. (**J**) phylogenetic position and stratigraphie of the Shinao/Yangjiachong/Xiaerhete ruminants (topology^2^). a stem Ruminantia, b *Archaeomeryx*, c *Chiyoumeryx* nov. gen. and *Krabimeryx gracilis*, d crown Ruminantia, e *Iberomeryx miaoi* nov. sp.; 1 lingual view, 2 occlusal view. Scale bare is 1 cm.

Figure 1A and Figure S1.

*v pars1982 *Lophiomeryx gracilis*—Miao: 532, Table 3, Figs. 6 and 7^20^.

v non1982 *Lophiomeryx gracilis*?—Miao: 536, Fig. 8^20^.

v pars1987 *L. gracilis*—Janis: 211^33^.

v pars*1997 L. gracilis*—Vislobokova: Fig. 3^21^.

v pars*2000 L. gracilis*—Guo, Dawson, and Beard: 247, Table 2^14^.

v pars*2001 L. gracilis*—Métais, Chaimanee, Jaeger, and Ducroq: 239, 241^17^.

v pars*2012 L. gracilis*—Mennecart: 62^34^.

### Neodiagnosis

*Krabimeryx gracilis* has an m2 that is wider than the m3; this is the other way round in *K. primitivus*. Moreover, the entoconid is less anterior relative to the hypoconid in *K. gracilis* than it is in *K primitivus*. The ectostylid is large in *K. gracilis*, while it is absent in *K. primitivus*. The cingulum on the upper molars in *K. gracilis* is more developed than in *K. primitivus*.

### Holotype

IVPP V 6546, partial skull with right and left M1–M3 (IVPP V 6546-1) and an associated right fragmented mandible with m2–m3 (IVPP V 6546-2) found in occlusion with the skull.

### Additional material

IVPP V 6549, right m3 on fragmented mandible; IVPP V 6550 left fragmented mandible with m1–m2; IVPP V 26638, right m1. Measurements are given in Table S1.

### Localities

Shinao Basin, Panxian County, Southwestern Guizhou, China; Xiaerhete locality, Jiminay County, Xingjiang, China. Late Eocene.

### Taxonomical attribution

The herein described specimens were first attributed to the genus *Lophiomeryx*^20^. However, the thorough reassessment of the specimens now leads to the conclusion that *Lophiomeryx gracilis *sensu Miao^20^ contains three different species and genera, but none of them can be assigned to *Lophiomeryx*.

Based on the presence of a strong lingual cingulum in upper molars and a short anteroposteriorly oriented postprotocrista, as well as the absence of a premetacristid and an anterior fossa widely open in the lower molars, we can conclude that the specimens, IVPP V 6546-1, IVPP V 6546-2, IVPP V 6549, and IVPP V 6550, belong to Lophiomerycidae or Tragulidae^35,36^. However, the absence of a large paraconid and the absence of an elongated external postmetacristid distinguish the specimens from primitive Tragulidae^17,36^. In *Zhailimeryx jingweni*, the cuspids are more slender than in the herein described specimens^14^, a feature the taxon shares with *K. primitivus*. In *Z. jingweni*, m1 and m2 are of relative similar width^14^, while in *K. primitivus* and the herein described specimens from Shinao the m2 is clearly bigger than the m1^17^. Similarly to *K. primitivus*, the herein described specimens differ from *Z. jingweni* in its lower molar lingual cusps being more laterally compressed, and in an entoconid that is slightly shifted to anterior with respect to the hypoconid, while it is more posterior in *Z. jingweni*^14,17^. Furthermore, *K. primitivus* and the herein described specimens from Shinao both lack the rudimentary paraconid present in *Z. jingweni*^14,17^.

Like *K. primitivus*, the here-described specimens differ from *Chiyoumeryx* nov. gen. (described below) and the *Lophiomeryx* species *L. mouchelini*, *L. chalaniati* and *L. angarae* by having more massive and more bunomorph lower molars^16,17,24,34,37^. Furthermore, *Zhailimeryx jingweni*, *K. primitivus*, and the herein described specimens differ from *Lophiomeryx* by the presence of a developed external postmetacristid and by a distinct entoconidian groove on the anterior side of the compressed entoconid^14,17^. In *Lophiomeryx*, the back fossa of m3 is widely open due to the strong reduction of the posthypoconulidcristid^34,37^. In contrast to this, *Krabimeryx primitivus* possesses a clearly developed posthypoconulidcristid forming a buckle on the m3 back basin^17^, similarly to the specimens from Shinao described here.

Summing up, the general morphology of the teeth in the herein described specimens is most similar to the one observed in *K. primitivus*. They both share a similar huge notch in lateral view between the third lobe of m3 and the entoconid and the entoconidian groove, features that clearly distinguishing them both from *Lophiomeryx* and *Zhailimeryx*. Thus, we attribute the specimens IVPP V 6546-1, IVPP V 6546-2, IVPP V 6549, and IVPP V 6550 to the genus *Krabimeryx*. However, significant differences occur with the type species, ruling out the synonymisation of *K. gracilis* nov. comb*.* and *Krabimeryx primitivus*. While both species are very similar in size, *K. primitivus* has an m3 wider than m2, while it is the converse for *K. gracilis* nov. comb*.* Moreover, the entoconid is less shifted to the anterior with respect to the hypoconid in *K. gracilis* nov. comb. than in *K primitivus*. There is no ectostylid in *K. primitivus*, while it is large in *K. gracilis* nov. comb., forming a transverse cristid between the protoconid and the hypoconid. The cingulum on the upper molars is more developed in *K. gracilis* nov. comb*.* than in *K. primitivus*.

Due to these differences we decided to create the new combination *Krabimeryx gracilis* nov. comb.

*Chiyoumeryx* nov. gen.

### ZooBank LSID

urn:lsid:zoobank.org:act:464C46E0-5A69-4AC1-A9DD-8A7DF76D5CC0.

### Etymology

Chiyou is a tribe leader of the ancient China, about 5–4 k years ago. Chiyou's tribe was believed to be in relation with the peoples in southern China; -*meryx* means ruminant in Greek.

### Diagnosis

*Chiyoumeryx* nov. gen. differs from *Zhailimeryx* and *Krabimeryx* notably by the absence of the entoconidian groove. The lower teeth are more laterally compressed in *Chiyoumeryx* nov. gen. and the metaconid is linguo-labiallly more central than in the two other genera. The posthypoconulidcristid in the lower molars of *Chiyoumeryx* nov. gen. is longer than in *Krabimeryx* and its p4 is posteriorly extended, while this part is reduced in *Krabimeryx*. *Chiyoumeryx* nov. gen. differs from *Lophiomeryx* by the shape of the mandible. In *Chiyoumeryx* nov. gen. there is no diastema between p1 and p2 and the diastema between c and p1 is extremely reduced. The outline of the mandible in occlusal view is relatively straight in this species. *Lophiomeryx* possesses a long diastema between c and p1 and a small one between p1 and p2, as well as a regularly curved occlusal outline of the corpus. The lower premolars of *Chiyoumeryx* nov. gen. are laterally compressed giving a more elongated aspect to these teeth than in *Lophiomeryx*. The trigonid is smaller than the talonid in m1 and m2 in *Chiyoumeryx* nov. gen. and the preprotocristid terminates centrally and does not reach the lingual side. In *Lophiomeryx* the trigonid and talonid are of similar size and the preprotocristid is longer and reaches the lingual side. Moreover, in *Chiyoumeryx* nov. gen., the posthypoconulidcristid is longer than in *Lophiomeryx*. The shape of the P4 in *Chiyoumeryx* nov. gen. differs from the one in *Lophiomeryx*: the posterolingual crista does not meet the posterolabial crista.

### Type species

*Chiyoumeryx* nov. gen. *shinaoensis* (Miao, 1982^20^).

### Included species

*Chiyoumeryx* nov. gen. *flavimperatoris* nov. sp.; ?*Chiyoumeryx* nov. gen. *turgaicus* (Flerow 1938^38^).

*Chiyoumeryx* nov. gen. *shinaoensis* (Miao, 1982^20^).

Figure 1B and Figure S2.

*v1982 *Lophiomeryx shinaoensis*—Miao: 530, Table 3, Figs. 3–5^20^.

v1987 *Lophiomeryx shinaoensis*—Janis: 203, 204, 211, 212, Fig. 8B^33^.

v*1997 Lophiomeryx shinaoensis*—Vislobokova: Fig. 3^21^.

v*2000 L. shinaoensis*—Guo, Dawson, and Beard: 247, Table 2^14^.

v*2001 L. shinaoensis*—Métais, Chaimanee, Jaeger, and Ducroq: 239–241, 241^17^.

v*2012 L. shinaoensis*—Mennecart: 62^34^.

### Neodiagnosis

*Chiyoumeryx* nov. gen. *shinaoensis* is bigger than *Chiyoumeryx* nov. gen. *flavimperatoris* nov. sp. but smaller than ?*Chiyoumeryx turagicus*. The transversely oriented anterior conid in the p4 in *Chiyoumeryx* nov. gen. *shinaoensis* differs from the obliquely oriented one in *Chiyoumeryx* nov. gen. *flavimperatoris* nov. sp*.* In *Chiyoumeryx* nov. gen. *shinaoensis*, the posterolingual conid is vestigial on p4. *Chiyoumeryx* nov. gen. *shinaoensis* has no anterior cingulid, while in *Chiyoumeryx* nov. gen. *flavimperatoris* nov. sp. there is a tiny anterior cingulid. *Chiyoumeryx* nov. gen. *shinaoensis* possesses lower crowns than ?*Chiyoumeryx* nov. gen. *turgaicus*. *Chiyoumeryx* nov. gen. *flavimperatoris* nov. sp. possesses an ectostylid, which is absent in ?*Chiyoumeryx* nov. gen. *turgaicus*.

### Holotype

IVPP V 6531, right mandible with p2–m3 and tooth socket of p1.

### Paratype

IVPP V 6532, right fragmented maxillary with P4–M3.

### Additional material

IVPP V 6533, right mandible with p2–m3 and tooth socket of i1–p1; IVPP V 6534, left fragments mandible with m1–m3; IVPP V 6535, right fragmented mandible with m1–m3; IVPP V 6536, left fragmented mandible with p4–m3; IVPP V 6537, right fragmented mandible with p4–m2; IVPP V 6538, left p4; IVPP V 6539, right maxillary with P3–M3; IVPP V 6540, right maxillary with P4–M2; IVPP V 6541, right maxillary with M2–M3; IVPP V 6542, left maxillary with P3–M1; IVPP V 6543, right maxillary with M1–M3; IVPP V 6544, Left M3; IVPP V 6545, left maxillary with P4–M3. Measurements are given in Table S1.

### Locality

Shinao Basin, Panxian County, Southwestern Guizhou, China. Late Eocene.

### Taxonomical attribution

Miao^20^ attributed the here described specimens to the genus *Lophiomeryx* assuming that these fossils belong to a traguloid. “*Lophiomeryx*” *shinaoensis* clearly is a Lophiomerycidae: anterior and posterior fossae are open on the lower molars due to the absence of a premetacristid and the extreme reduction or absence of a postentocristid, there is no external postprotocristid, there is a mesolingual conid on the p4, the symphysis of the mandible extends backward up to the p1^2,36^. It also shares with undisputable Lophiomerycidae a reduced posthypoconulidcristid that does not enclose the third lobe lingually.

*“Lophiomeryx” shinaoensis* differs from *Zhailimeryx* and *Krabimeryx* in the absence of the entoconidian groove^14,17^. Moreover, the teeth are more laterally compressed in *“Lophiomeryx” shinaoensis* and the metaconid is linguo-labially more centeral^14,17^. The posthypoconulidcristid in *“Lophiomeryx” shinaoensis* is more elongated than in *Krabimeryx* and its p4 has an extended posterior part, while it is reduced in *Krabimeryx*^17^.

Contrary to what was suggested by Métais and Vislobokova^2^, *Miomeryx altaicus*^24^ is currently known only by its holotype, which is an upper tooth row (AMNH 20383, see Matthew and Granger^24^). Comparable to *M. altaicus*, the postprotocrista reaches the premetaconulecrista on the M2 in *“Lophiomeryx” shinaoensis*. These two cristae fuse totally on the M3 in the here described specimens. However, even if both genera also bear a very strong cingulum, *“Lophiomeryx” shinaoensis* clearly differs from *M. altaicus* in having broader and squarer molars and straighter lingual cristae in the P4.

Miao^20^ compared the here revised fossils with the seven *Lophiomeryx* species considered valid at that time. Unfortunately, very few specimens document most of these species and there is considerable doubt considering the genus attribution of most of them^34,36–39^. In any case, we agree with Miao^20^ (p. 535) that “*L.* [= *Praetragulus*] *gobiae* is readily distinguished from other known *Lophiomeryx* species as well as from *L. shinaoensis* by the absence of p1, the anterior flange of metaconid joining protoconid crescent.”. Miao^20^ (p. 535) already noticed that “*Lophiomeryx chalaniati*, *Lophiomeryx gaudry* [= *Iberomeryx minor*], and *Lophiomeryx benarensis* are radically different from the present specimens in the anterior branches of the protoconid crescent [= preprotocristid], of m1 and m2 not reaching the lingual border while the posterior branches of hypoconid crescent [= posthypocristid], doing so”. *“Lophiomeryx” shinaoensis* shares this condition with the Mongolian *Lophiomeryx angarae*^24^. However, the trigonid is smaller than the talonid on m1 and m2 in *“Lophiomeryx” shinaoensis* and the preprotocristid ends in the labio-lingual axis of the molars, while trigonid and talonid are of more similar width combined with a longer preprotocristid in the European *Lophiomeryx* species and *L. angarae*^16,34,37^. The shape of the P4 in *“Lophiomeryx” shinaoensis* is very different from *Lophiomeryx* (see Brunet and Sudre^37^, Figs. 4 and 6). In *Lophiomeryx*, the posterolingual crista fuses with the posterolabial crista. In *“Lophiomeryx” shinaoensis*, the curved posterolingual crista does not join the distal end of the posterolabial crista but reaches the labial side. Furthermore, *“Lophiomeryx” shinaoensis* clearly differs from *L. angarae L. mouchelini*, and *L. chalaniati* in the shape of the mandible. These three species of *Lophiomeryx* possess a very elongated diastema between c and p1 and a small one between p1 and p2^24,36,37^. As part of the genus diagnosis, Mennecart^34^ (p. 62 and p. 67), adapted from Brunet and Sudre^37^ and Métais and Vislobokova^2^, noticed that “the *corpus mandibulae* presents [in *Lophiomeryx*: *L. angarae*, *L. mouchelini*, and *L. chalaniati*^24,34,37^] a concave ventral profile just behind the mandible symphysis, then it becomes regularly convex until the beginning of the ramus, where there is a rounded *incisura vasorum*. […] On the anterior part of the mandible there are two *foramen mentale*.” Moreover he wrote that the “p1 is always reduced and leaf-like, separated from c and p2 by diastemata.” (Mennecart^34^, p. 67). In *“Lophiomeryx” shinaoensis* there is no diastema between p1 and p2 and the diastema between c and p1 is extremely reduced. The p1 is relatively big considering the root size. The lower outline of the mandible in lateral view is relatively straight. *“Lophiomeryx” shinaoensis* shares these characteristics with “*Lophiomeryx*” *turgaicus*^40^. Miao^20^ (p. 535) already noticed strong similarities between “*Lophiomeryx*” *turgaicus* and *“Lophiomeryx” shinaoensis*. The lower premolars of “*Lophiomeryx*” *turgaicus* and *“Lophiomeryx” shinaoensis* are strongly laterally compressed and the p4 is rectangular, giving the lower premolar toothrow an more elongated aspect than in *L. angarae*, *L. mouchelini*, and *L. chalaniati*^20,24,30,38,40^. Moreover, in these two species, the posthypoconulidcristid is of similar length, longer than in *L. angarae*, *L. mouchelini*, and *L. chalaniati*.

Based on these observations, we can assume that *“Lophiomeryx” shinaoensis* and “*Lophiomeryx*” *turagicus* cannot be assigned to the genus *Lophiomeryx* and may both belong to the same new Lophiomerycidae genus that we here name *Chiyoumeryx* nov. gen. *Chiyoumeryx* nov. gen. *shinaoensis* differs from ?*Chiyoumeryx* nov. gen. *turgaicus* nov. comb. in being lower crowned, smaller, possessing an ectostylid, having the symphysis starting under p1, and a shorter diastema.

*Chiyoumeryx* nov. gen. *flavimperatoris* nov. sp.

Figure 1C and Figure S3.

v1961 cf. *Miomeryx* sp.—Xu: 316, 323, 324^26^.

v pars1982 *Lophiomeryx gracilis*—Miao: 532, Table 3, Fig. 9a,b^20^.

v non1982 *Lophiomeryx gracilis*?—Miao: 536, Fig. 8^20^.

*1983 Lophiomeryx* sp.—Wang & Zhang: 122, 127^41^.

v*1983* cf. *Miomeryx* sp.—Wang & Zhang: 123^41^.

v*1997 Miomeryx* sp.—Vislobokova: Fig. 3^21^.

v pars*1997 L. gracilis*—Vislobokova: Fig. 3^21^.

v*1999* cf. *Miomeryx* sp.—Zhang, Long, Ji, & Ding: 7, Table 5^27^.

v pars*2000 L. gracilis*—Guo, Dawson, and Beard: 247, Table 2^14^.

v pars*2001 L. gracilis*—Métais, Chaimanee, Jaeger, and Ducrocq: 239, 241^17^.

v*2007 Miomeryx* sp.—Métais and Vislobokova: 194^2^.

v pars*2012 L. gracilis*—Mennecart: 62^34^.

### ZooBank LSID

urn:lsid:zoobank.org:act:1DF6F58C-F08B-4657-BD4A-7C597653926F.

### Etymology

meaning yellow (*flavor-*) emperor (*imperatoris*) in latin. Chiyou fought with the Yellow Emperor, the ancestor of Chinese, but was defeated.

### Diagnosis

*Chiyoumeryx* nov. gen. *flavimperatoris* nov. sp. shows the above-mentioned characteristics of the genus. *Chiyoumeryx* nov. gen. *flavimperatoris* nov. sp. is smaller than *Chiyoumeryx* nov. gen. *shinaoensis* and ?*Chiyoumeryx* nov. gen. *turgaicus*. The p4 of *Chiyoumeryx* nov. gen. *flavimperatoris* nov. sp. differs from *Chiyoumeryx* nov. gen. *shinaoensis* by an oblique anterior conid, which is labio-lingually oriented in the larger species. A very short posterolingual conid is located between the posterolabial cristid and the transverse cristid in the p4 of *Chiyoumeryx* nov. gen. *flavimperatoris* nov. sp., while it is absent on *Chiyoumeryx* nov. gen*. shinaoensis*. In *Chiyoumeryx* nov. gen. *flavimperatoris* nov. sp., there is a tiny anterior cingulid, while it is absent in *Chiyoumeryx* nov. gen. *shinaoensis*.

### Holotype

IVPP V 6547, right mandible with p4–m3 (previously attributed to *Lophiomeryx gracilis*^20^).

### Paratype

IVPP V 6548, left mandible with p4–m3 (previously attributed to *Lophiomeryx gracilis*^20^).

### Additional material

IVPP V 2600, left p4–m2 (previously attributed to cf. *Miomeryx* sp.^26^). Measurements are given in Table S1.

### Localities

Yangjiachong locality lying in the Caijiachong marls, Qujing, Yunnan, China; Shinao Basin, Panxian County, Southwestern Guizhou, China. Late Eocene.

### Taxonomical attribution

IVPP V 6547 and IVPP V 6548 from Shinao were previously attributed to *Lophiomeryx gracilis*^20^, while IVPP V 2600 from Caijiachong marls was first described as cf. *Miomeryx* sp.^26^. All these specimens share the same size and dental morphology, and originate from a similar stratigraphic position. That is why we attribute them to the same species.

None of these specimens can be attributed to *Krabimeryx* or *Zhailymeryx,* as the entoconidian groove is absent^14,17^. Furthermore, the external postmetacristid is more marked in the considered specimens than in *Krabimeryx* and *Zhailymeryx*, forming a deep groove. The third basin is also very different in the here-described specimens from *Krabimeryx* and *Zhailymeryx*: the third lobe is a little tilted parallel with the prehypoconulidcristid and posthypoconulidcristid. The back fossa of m3 is very narrow.

Furthermore, the here-described specimens can be distinguished from *K. gracilis* (previously attributed to the same species), by a smaller size and a slenderer shape. The ectostylid is smaller than in *K. gracilis*. The anterior cingulid in the lower molars is stronger in *K. gracilis* than in the here-considered specimens. The small postentocristid (especially on m3) of the here-described specimens is absent in *K. gracilis*.

The here-described specimens possess all characteristics in the lower molars that are typical for *Chiyoumeryx* nov. gen. and distinguish this genus from *Lophiomeryx*^24,34,37^. Furthermore, as in *Chiyoumeryx* nov. gen. *shinaoensis*, the p4 is laterally compressed giving it a more elongated aspect than in *Lophiomeryx*^24,34,37^. Therefore, we consider it justified assigning the here-described specimens to *Chiyoumeryx* nov. gen. However, they differ from *Chiyoumeryx* nov. gen. *shinaoensis* in as smaller size and the morphology of the p4: (1) the anterior conid is oblique while it is labio-lingually oriented in *Chiyoumeryx* nov. gen. *shinaoensis*. (2) There is a tiny anterior cingulid that is absent in *Chiyoumeryx* nov. gen. *shinaoensis*. (3) There is no additional cristid on the mesolingual conid, which is a well-rounded conid, while in *Chiyoumeryx* nov. gen. *shinaoensis*, there is a short posterolingual cristid. (4) The posterolingual conid stands between the posterolabial cristid and the transverse cristid, while in *Chiyoumeryx* nov. gen. *shinaoensis*, the posterolingual conid is very small and oblique between the transverse cristid and the posterior stylid and does not join the posterolabial cristid. Due to these distinct differences we erect a new species: *Chiyoumeryx* nov. gen. *flavimperatoris* nov. sp.

Family Tragulidae Milne-Edwards, 1864^42^.

Genus *Iberomeryx* Gabunia, 1964^43^.

### Diagnosis (modified from Mennecart et al.^36^)

Small-sized ruminant with upper molars possessing the following combination of characters: well-marked parastyle and mesostyle in small-column shape; strong paracone rib; metacone rib absent; metastyle absent; unaligned external walls of metacone and paracone; strong postprotocrista stopping against the anterior side of the premetaconulecrista; continuous lingual cingulum, stronger under the protocone. Lower dental formula is primitive (3–1–4–3) with non-molarized premolars. Tooth c is adjacent to i3. Tooth p1 is single-rooted, reduced and separated from c and p2 by a short diastema. The premolars have a well-developed anterior conid. Teeth p2–p3 display a distally bifurcated mesolabial conid. Tooth p3 is the largest premolar. Tooth p4 displays no mesolingual conid and a large posterior valley. Regarding the lower molars, the trigonid and talonid are lingually open with a trigonid more tapered than the talonid. The anterior fossa is open, due to a forward orientation of the preprotocristid and the presence of a paraconid. The internal postprotocristid is oblique and the external postprotocristid reaches the prehypocristid. The internal postprotocristid, postmetacristid and preentocristid are fused and Y-shaped. Protoconid and metaconid display a weak *Tragulus* fold and a well-developed *Dorcatherium* fold, respectively. The mandible displays a regularly concave ventral profile in lateral view, a marked incisura vasorum, a strong mandibular angular process, a vertical ramus, and a stout condylar process.

### Type species

*Iberomeryx parvus* Gabunia, 1964^43^ from Benara (Georgia), late Oligocene^44^.

### Included species

*I. minor*^45^, *Iberomeryx miaoi* nov. sp.

*Iberomeryx miaoi* nov. sp.

Figure 1D and Figure S4.

v 1982 *Lophiomeryx gracilis*?—Miao: 536, Fig. 8^20^.

### ZooBank LSID

urn:lsid:zoobank.org:act:EE3F88E9-0EAF-4EC6-A46F-8623241E614B.

### Diagnosis

*Iberomeryx* with a very large paraconid, which is smaller in *Iberomeryx minor* and *Iberomeryx parvus*. The metastylid is not strong but is more developed than in the other species. The ectostylid is big on m1, smaller on m2 and absent on m3, while *I. minor* displays an ectostylid on all molars and *I. parvus* none at all. *Iberomeryx miaoi* nov. sp. is of similar size to *I. minor* and its m2 is smaller than the one of *I. parvus*. It differs from *I. minor* by a thin anterior cingulid. Moreover, its protoconid is positioned slightly more anterior than in *I. parvus*. The molars appear to be more massive and bulkier in this species than in *I. minor* and *I. parvus.*

### Holotype

IVPP V 6551, left mandible with m1–m3 (only specimen known). m1 5.1 × 3.5, m2 5.2 × 4.1, m3 8.0 × 4.0.

### Etymology

We dedicate this species to Prof. Miao Desui who was the first to describe the Shinao fauna.

### Locality and horizon

Shinao Basin, Panxian County, Southwestern Guizhou, China. Late Eocene.

### Taxonomical attribution

This minute ruminant was referred to *Lophiomeryx gracilis*? by Miao^20^. However, he already noticed that the size of this individual was smaller than in the other specimens attributed to *Lophiomeryx gracilis*. Miao^20^ excluded an attribution of IVPP V 6551 to "*Lophiomeryx" gaudryi* due to a closed posterior section of the posterior fossa on the m3. However, in both teeth, the posterior fossa is still open by the reduction of the postentocristid.

The here-described specimen clearly differs from *Lophiomeryx* by the presence of an external postmetacristid forming a slight *Dorcatherium* fold, a developed external postprotocristid (clearly visible at least on m2), and a large paraconid^36^. Furthermore the external postprotocristid and prehypocristid are connected on their distal ends and the third basin of m3 forms a well-formed buckle, unlike the condition in Lophiomerycidae^14,16,33,36,37^. The combination of these characters is typical for Tragulidae^36^.

Very few taxa are so far known in the early evolution of the Tragulidae. Only *Archaeotragulus*, *Iberomeryx*, and *Nalameryx* are recognized as potential Paleogene Tragulidae^17,36,46^, of which *Archaeotragulus* is currently the oldest representative described^17,47^. *Archaeotragulus* possesses lower molars with a broadened talonid in comparison to the trigonid and displays an entoconidian groove^36^. In the case of IVPP V 6551, the trigonid and talonid are of similar size and no specific entoconidian groove can be observed. Mennecart et al.^36^ considered *Nalameryx* a Tragulidae notably based on the presence of the M structure (the external postmetacristid, the internal postmetacristid, the internal postprotocristid, and the external postprotocristid are interconnected forming a M in occlusal view), including the *Tragulus* fold and *Dorcatherium* fold, and the absence of a rounded mesolingual conid in the p4^35^. IVPP V 6551 differs from *Nalameryx* in having an m3 wider than m1 and similar m1 and m2 widths^17^. In size proportions and molar morphology, IVPP V 6551 resembles the genus *Iberomeryx*. In IVPP V 6551, the relative size of the m2 is more similar to *I. minor*. In *Iberomeryx minor*, the anterior cingulid is big^36,46^, while in *Iberomeryx parvus* the cingulid is thin^48^ like in IVPP V 6551. The teeth of IVPP V 6551 appear to be more massive and bulkier than in *I. minor* and *I. parvus*^36,48^. Similarly to *I. minor*, the protoconid of IVPP V 6551 is a little more anterior than in *I. parvus*^36,48^. IVPP V 6551 clearly differs from *I. parvus* and *I. minor* by the presence of a very large paraconid, which is smaller in the two other species^36,48^. Moreover, the metastylid in IVPP V 6551 is slightly more developed than in *I. minor* and not present in *I. parvus*^43,48^. *Iberomeryx minor* displays an ectostylid on all molars^36^, while this structure is absent from *I. parvus*^48^. The ectostylid in IVPP V 6551 is large on m1 to absent on m3. Based on these differences we decided to erect the new species *Iberomeryx miaoi* nov. sp.

### Origin of crown Ruminantia and dispersal pattern of Paleogene Eurasian ruminants

So far five families and 13 genera of Ruminantia are known during the middle and late Eocene in Eurasia^2,18,19^. Based on molecular data, the origin of crown ruminants should be searched for between the latest late Paleocene (56.5 Ma) and the latest early Oligocene (29 Ma)^49,50^. With the description of stem Tragulidae from the early Oligocene of Western Europe (*Iberomeryx*) and the late Eocene from Southern Thailand (*Archaeotragulus*)^17^, Mennecart et al.^26^ and Mennecart and Métais^51^ verified that the oldest crown ruminants date back at least to the latest Eocene (34 Mya). The presence of the tragulid genus *Iberomeryx* in Shinao, Southern China, further confirms this and may actually represent the oldest fossil of a Tragulidae known and thus of a crown Ruminantia (37–35 Mya, Fig. 1), since no Pecora is known during the Eocene so far^51^.

**Figure 2:**
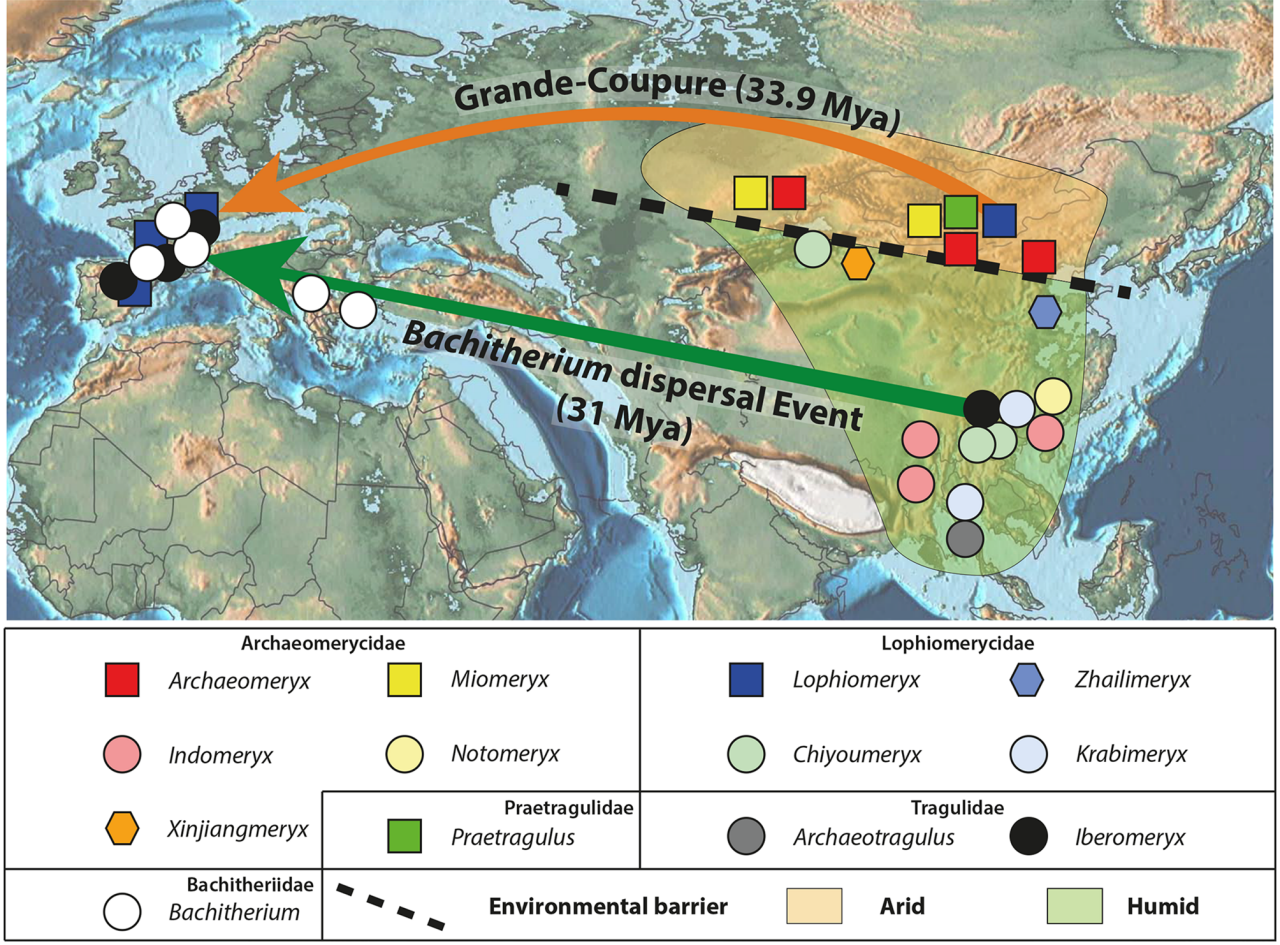
Paleobiogeography of the Eurasiatic ruminants during the Eocene at the genus level. The localities are from the synthesis of data^2,17,18,22,49^. The palinspastic map is modified from Scotese^52^.

The here presented reassesment of the Shinao ruminants in combination with literature data reveals a clear pattern in the distribution of Eocene ruminants. Among Archaeomerycidae, *Archaeomeryx* and *Miomeryx* are found in Northern and Central Asia [Kazhakstan, Mongolia, and northern part of China^2,21,53^ (see Fig. 2)]. The lophiomerycid *Lophiomeryx* (as *Lophiomeryx angarae*) as well as the Asiatic Praetragulidae (*Praetragulus*) occupy the same area^2^. The Mongolian *Lophiomeryx angarae* is most likely closely related to the European species *Lophiomeryx mouchelini*. Due to the strong morphological similarities, some specimens of *L. mouchelini* were actually first described as *Lophiomeryx* cf. *angarae*^54^. *Lophiomeryx mouchelini* or its ancestors arrived in Europe with the Grande-Coupure dispersal event at the Eocene–Oligocene transition ca. 34 Mya ago (oldest European records: Calaf, Spain, MP22; Möhren 9, Germany, MP21-22; age comprised between the German localities Haag2 MP21 and Möhren 13 MP22^34,37,53^). The close relationship of these European and the Mongolian species confirms that the origin of the Grande-Coupure cohort may be deeply anchored in the Eocene of Central-Northern Asia (Fig. 2).

The Southern part of Asia presents a totally different ruminant community at the genus level and includes the Archaeomerycidae *Indomeryx* and *Notomeryx*, the Lophiomerycidae *Krabimeryx* and *Chiyoumeryx* nov. gen., the Bachitheriidae *Bachitherium* and the Tragulidae *Archaetrogulus* and *Iberomeryx*^2,17–19,21,53^ (see Fig. 2). The oldest *Bachitherium* is currently known from the Balkan area during the Eocene^18,19^. The Tethys Ocean separated this area from Western Europe until its progressive disappearance during the Oligocene, ca. 31 Mya^55,56^. *Bachitherium* and a cohort of rodents (*Pseudocricetodon*, *Paracricetodon*, and the Melissodontinae)^19^ did not reach Western Europe prior to the opening of this passage. Similarly to the genus *Bachitherium*, *Iberomeryx* arrived in Western Europe after the drying out of the Tethys Ocean ca. 31 Mya, during the *Bachitherium* dispersal event^18,19,36^. *Iberomeryx* is mainly known from the middle early Oligocene of Western Europe^34,36,57^ and the late Oligocene of Anatolia and Georgia^43,48,58^. Discovering *Iberomeryx* in the Eocene of Eastern Asia confirms an Asiatic origin of this genus. The close relationship between South-eastern Europe and South-eastern Asia is furthermore supported by anthracotheriids (extinct artiodactyls related to hippopotamids) and rhinocerotoids^59,60^.

Mennecart et al.^18,19^ proposed that mammals originating from Asia arrived in Western Europe during the early Oligocene in two faunal events: the Grande-Coupure, ca. 33.9 Mya and the *Bachitherium* dispersal event, ca. 31 Mya. These two faunal events imply two different and diachronous ways of dispersal. The fact that Eocene taxa from South-eastern Asia did not arrive in Western Europe prior to 31 Mya indicates that the *Bachitherium* dispersal Event cohort might be deeply anchored in the Eocene of Southern Asia (Fig. 2), while genera recorded from the Eocene of Central Asia are known to have arrived already during the Grande-Coupure and thus originated from a different palaeobiogeographic province. The Grande-Coupure was a dispersal event using a Northern way over the closed Turgai Strait and probably originating from Central Asia (Fig. 2). The *Bachitherium* dispersal event is a stepwise story with a first dispersion from Southern Asia to South-eastern Europe along the Southern path (Fig. 2) and then the dispersal throughout Europe thanks to the closure of the Tethyian Ocean^18,19^.

The south-eastern part of Asia has shown very few changes from a warm and humid climate and environment since the Eocene^4^, while Northern Asia underwent a transition from warm and humid subtropical environments during the Eocene to steppe environments in the Pliocene, e.g.^3–5^. In this light it is not surprising that an increasing number of paleontological and geological studies indicate that Asia had already experienced a strong latitudinal environmental zonation during the middle and the late Eocene, e.g.^6,13^.

These different climatic and environmental conditions in Central and South Asia led to two distinct palaeobiogeographical provinces clearly traceable in assemblages of herbivores like ruminants that was already apparent during the Eocene. The Central Asian ruminants were living in a more arid environment than the ones from South-eastern Asia (see Fig. 2). The tropical and wet environments from the South-eastern Asia led to the emergence of the Tragulidae (*Iberomeryx* and *Archaeotragulus*) and of the anthracotheriids.

## Materials and methods

### Materials

The fossils are housed at the Institute of Vertebrate Paleontology and Paleoanthropology of the Chinese Academy of Sciences in Beijing. Precise descriptions and measurements of the species can be found in Supplementary data 1.

### Methods

Measurements have been realized thanks to a calliper (precision 0.2 mm) and can be found in Table S1. The dental nomenclature was modified after Bärmann and Rössner^61^ (see Fig. 1).

Conventional abbreviations used in front of the year in the synonymy list follow Matthews^62^: * = the work validates the species; v = the authors have seen the original material of the reference; pars = the reference applies only in part to the species under discussion; non = the reference actually does not belong to the species under discussion; no sign = the authors were unable to check the validity of the reference. Years in italics indicate a work without description or illustration.

### Abbreviations

IVPP, Institute of Vertebrate Paleontology and Paleoanthropology of the Chinese Academy of Sciences in Beijing (China); AMNH, American Museum of Natural History (USA). p/P, lower/upper premolar’ m/M, Lower/upper molar.

### Nomenclatural acts

This published work and the nomenclatural acts it contains, have been registered in ZooBank:

- *Chiyoumeryx* nov. gen.: 464C46E0-5A69-4AC1-A9DD-8A7DF76D5CC0.
- *Chiyoumeryx* nov. gen. *flavimperatoris* nov. sp.: 1DF6F58C-F08B-4657-BD4A-7C597653926F.
- *Iberomeryx miaoi* nov. sp.: EE3F88E9-0EAF-4EC6-A46F-8623241E614B.

## Supplementary Information

Supplementary Information.
