# Author Correction: Ruminants reveal Eocene Asiatic palaeobiogeographical provinces as the origin of diachronous mammalian Oligocene dispersals into Europe

**DOI:** 10.1038/s41598-021-98946-1

**Published:** 2021-09-21

**Authors:** Bastien Mennecart, Manuela Aiglstorfer, Yikun Li, Chunxiao Li, ShiQi Wang

**Affiliations:** 1 Naturhistorisches Museum Wien, Burgring 7, 1010 Vienna, Austria; 2 Naturhistorisches Museum Basel, Augustinergasse 2, 4001 Basel, Switzerland; 3 Naturhistorisches Museum Mainz/Landessammlung Für Naturkunde Rheinland-Pfalz, Reichklarastraße 10, 55116 Mainz, Germany; 4 Center for Research and Education on Biological Evolution and Environment, Nanjing University, Nanjing, 210023 China; 5 Key Laboratory of Vertebrate Evolution and Human Origins of Chinese Academy of Sciences, Institute of Vertebrate Paleontology and Paleoanthropology, Chinese Academy of Sciences, Beijing, 100044 China; 6 CAS Center for Excellence in Life and Paleoenvironment, Beijing, 100044 China

Correction to: *Scientific Reports* 10.1038/s41598-021-96221-x, published online 06 September 2021

In the original version of this Article, ShiQi Wang was omitted as a corresponding author. Correspondence and requests for materials should also be addressed to wangshiqi@ivpp.ac.cn.

The original Article has been corrected.

