# Supplementary material for: Ruminants reveal Eocene Asiatic palaeobiogeographical provinces as the origin of diachronous mammalian Oligocene dispersals into Europe
Source: Sci Rep. 2021 Sep 6;11:17710. doi: 10.1038/s41598-021-96221-x (PMC8421421; doi:10.1038/s41598-021-96221-x)
Supplement: Supplementary file 1 — Supplementary Information. [file 41598_2021_96221_MOESM1_ESM.zip › Supplementary data 1/Supplementary Data_Mennecart et al_Eocene China.docx]

**Supplementary Data**

**SYSTEMATIC PALAEONTOLOGY**

Mammalia Linnaeus, 1758

Artiodactyla Owen, 1848

Ruminantia Scopoli, 1777

Infraorder Tragulina Flower, 1883

Family Lophiomerycidae Janis, 1987

**Included genera.** *Lophiomeryx*, *Zhailimeryx*, *Krabimeryx*, *Chiyoumeryx* nov. gen.

Genus *Krabimeryx* Métais, Chaimanee, Jaeger, and Ducroq, 2001

**Etymology**. *Krabi-* from Krabi Basin, where the fossils were found, and –*meryx* is the Greek word for ruminant.

**Diagnosis [modified after Métais et al. 2001].** Small primitive ruminant with lower molars morphologically close to those of *Zhailimeryx*. *Krabimeryx* differs from *Zhailimeryx* in: more laterally compressed lingual cuspids in the lower molars; an entoconid displaced to anterior with respect to the hypoconid; the lack of both a paraconid and a hypoconulid in m1 and m2; a p4 with a mesolingual conid that is located more posterior and less individualized; a p4 without a distinct posterolingual conid. *Krabimeryx* differs from *Lophiomeryx* by less selenodont labial cuspids in the lower molars, the presence of a developed external postmetacristid, and by a distinct groove on the anterior side of the entoconid, the entoconidian groove. *Krabimeryx* can be distinguished from *Iberomeryx* in having a well-marked entoconidian groove; the lack of a clear external postprotocristid; the third lobe of m3 not forming a complete buckle; and a more transversely compressed hypoconulid in the m3. *Krabimeryx* possesses a huge notch in lingual view between the entoconid and the third lobe in the m3.

**Type species.** *Krabimeryx primitivus* Métais, Chaimanne, Jaeger, and Ducroq, 2001.

**Included species.** *Krabimeryx gracilis* nov. comb. (Miao, 1982)

*Krabimeryx gracilis* nov. comb. (Miao, 1982)

Fig. S1

*v pars 1982 *Lophiomeryx gracilis*—Miao: 532, tab. 3, fig. 6-7

v non 1982 ?*Lophiomeryx gracilis* —Miao: 536, fig.8

v pars 1987 *L. gracilis*—Janis: 211

v pars *1997* *L. gracilis*—Vislobokova: fig. 3

v pars *2000* *L. gracilis*—Guo, Dawson, and Beard: 247, tab. 2

v pars *2001* *L. gracilis*—Métais, Chaimanee, Jaeger, and Ducroq: 239, 241

v pars *2012* *L. gracilis*—Mennecart: 62

**Diagnosis [revised and modified after Miao, 1982 ].** *Krabimeryx gracilis* has an m2 that is wider than the m3; this is the other way round in *K. primitivus*. Moreover, the entoconid is less anterior relative to the hypoconid in *K. gracilis* than it is in *K primitivus*. The ectostylid is large in *K. gracilis*, while it is absent in *K. primitivus*. The cingulum on the upper molars in *K. gracilis* is more developed than in *K. primitivus*.

**Holotype.** IVPP V 6546, partial skull with right and left M1-M3 (IVPP V 6546-1) and an associated right fragmented mandible with m2-m3 (IVPP V 6546-2) found in occlusion with the skull.

**Additional material.** IVPP V 6549, right m3 on fragmented mandible; IVPP V 6550 left fragmented mandible with m1-m2; IVPP V 26638, right m1.

**Localities.** Shinao Basin, Panxian County, Southwestern Guizhou, China; Xiaerhete locality, Jiminay County, Xingjiang China. Late Eocene.

**Description**

Lower tooth row: m2 is wider than m3. The trigonid is broadly open anteriorly due to the reduced or absent premetacristid (only on fresh teeth there is an extremely small premetacristid located on the apex of the metaconid). The preprotocristid is strong and reaches the lingual side in fresh teeth. There is no external postprotocristid in the lower molars. The metaconid is located posterior in the trigonid and terminates at the level of the perpendicularly oriented internal postprotocristid. The metaconid's anterior part is well-rounded. There are two postmetacristids. The external postmetacristid is sweak. The internal one is relatively transverse and joins the postprotocristid forming a triple junction with the straight and elongated preentocristid. The entoconid is slightly shifted to anterior in comparison to the hypoconid.The entoconid is slightly linguo-labially compressed with a well-marked entoconidian groove, especially on m1 and m2. There is no postentocristid and the posterior basin is open. The long prehypocristid almost, but not fully, reaches the junction of postmeta- (internal), postproto- and preentocristid. The posthypocristid does not reach the lingual side of the molars and terminates on the posterolabial face of the entoconid in m1 and m2. The ectostylid is big. It deeply intrudes the valley between the proto- and the hypoconid. On m1 and m2, it forms a big triangular structure in occlusal view. In the m3 it forms a cristid like structure oriented perpendicular to the length axis of the tooth and strongly protrudes in between proto- and hypoconid. The anterior and posterior cingulids are big on m1 and m2. The anterior one is even a little anteriorly protruding on fresh teeth. The hypoconulid forms the distal part of the tooth in the m3. It is centrally located in the main axis of the m3. The third lobe of m3 forms a buckle. The back fossa is slightly oblique, enlarged, and open anterolingually, as the posthypoconulidcristid does not reach the entoconid. Both hypoconulidcristids are curved forming a rounded back fossa of m3.

Upper tooth row: The size increases from M1 to M3. They are square; even the M3 since the metaconule is not reduced. The lingual cusps are aligned. The paracone bears a labial rib very globular, while the metacone is flattened on its labial wall. The anterior fossa is wide. Except for the premetaconulecrista, which is a little curved, all cristae are relatively straight. The postparacrista and the premetacrista are fused with the mesostyle. The postparacrista reaches the mesostyle on its lingual part and the premetacrista on its posterolingual side, making it salient in occlusal view. The postmetacrista seems to be slightly longer than the other labial cristae. It terminates in the metastyle. The postprotocrista is posterolabially oriented and terminates basally in the central part of the premetaconulecrista. The very elongated and slightly curved premetaconulecrista intrudes deeply between the paracone and the metacone, almost reaching the mesostyle. The postmetaconulecrista fuses with the base of the metastyle enclosing the posterior fossa. On M3, the premetaconulecrista bears on its posterior half an additional “cone” (see 14 in Figure 2H), and the anterior fossa has two enamel spurs: one at the lingual base of the paracone and one in its posterior-lingual part. There is neither bifurcation of the postprotocrista nor a metaconule fold. The parastyle, the paracone rib, the metacone rib, and the metastyle are in line. The mesostyle is projecting labially and is big and rounded. It becomes less globular from M1 to M3. The parastyle is big and globular, and as big as the mesostyle on the M2. The size of the metastyle increases from M1 to M3. There is a big anterolingual cingulum surrounding the protocone. In the M1 it continues surrounding the metaconule. In M2 and M3 it becomes weaker and is interrupted at the lingual wall of the metaconule. The entostyle is big. Its shape is a relatively labio-lingually oriented crista. It connects cingulum and distal part of the postprotocrista in the M2. On the M3, it forms a complex and large structure comprising three elongated enamel spurs. The main one is fused with base of the central part of the postprotocrista.

*Chiyoumeryx* nov. gen.

**ZooBank LSID.** urn:lsid:zoobank.org:act:464C46E0-5A69-4AC1-A9DD-8A7DF76D5CC0

**Etymology.** Chiyou is a tribe leader of the ancient China, about 5k-4k years ago. Chiyou's tribe was believed to be in relation with the peoples in southern China; -*meryx* means ruminant in Greek.

**Diagnosis.** *Chiyoumeryx* nov. gen. differs from *Zhailimeryx* and *Krabimeryx* notably by the absence of the entoconidian groove. The lower teeth are more laterally compressed in *Chiyoumeryx* nov. gen. and the metaconid is linguo-labiallly more central than in the two other genera. The posthypoconulidcristid in the lower molars of *Chiyoumeryx* nov. gen. is longer than in *Krabimeryx* and its p4 is posteriorly extended, while this part is reduced in *Krabimeryx*. *Chiyoumeryx* nov. gen. differs from *Lophiomeryx* by the shape of the mandible. In *Chiyoumeryx* nov. gen. there is no diastema between p1 and p2 and the diastema between c and p1 is extremely reduced. The outline of the mandible in occlusal view is relatively straight in this species. *Lophiomeryx* possesses a long diastema between c and p1 and a small one between p1 and p2, as well as a regularly curved occlusal outline of the corpus. The lower premolars of *Chiyoumeryx* nov. gen. are laterally compressed giving a more elongated aspect to these teeth than in *Lophiomeryx*. The trigonid is smaller than the talonid in m1 and m2 in *Chiyoumeryx* nov. gen. and the preprotocristid terminates centrally and does not reach the lingual side. In *Lophiomeryx* the trigonid and talonid are of similar size and the preprotocristid is longer and reaches the lingual side. Moreover, in *Chiyoumeryx* nov. gen., the posthypoconulidcristid is longer than in *Lophiomeryx*. The shape of the P4 in *Chiyoumeryx* nov. gen. differs from the one in *Lophiomeryx*: the posterolingual crista does not meet the posterolabial crista.

**Type species.** *Chiyoumeryx* nov. gen. *shinaoensis* (Miao, 1982)

**Included species.** *Chiyoumeryx* nov. gen. *flavimperatoris* nov. sp.; ?*Chiyoumeryx* nov. gen. *turgaicus* (Flerow 1938).

*Chiyoumeryx* nov. gen. *shinaoensis* (Miao, 1982)

Fig. S2

*v 1982 *Lophiomeryx shinaoensis*—Miao: 530, tab. 3, fig. 3-5

v 1987 *Lophiomeryx shinaoensis*—Janis: 203, 204, 211, 212, fig. 8B

v *1997* *Lophiomeryx shinaoensis*—Vislobokova: fig. 3

v *2000* *L. shinaoensis* —Guo, Dawson, and Beard: 247, tab. 2

v *2001* *L. shinaoensis*—Métais, Chaimanee, Jaeger, and Ducroq: 239-241, 241

v *2012* *L. shinaoensis*—Mennecart: 62

**Neodiagnosis.** *Chiyoumeryx* nov. gen. *shinaoensis* is bigger than *Chiyoumeryx* nov. gen. *flavimperatoris* nov. sp. but smaller than ?*Chiyoumeryx* *turagicus*. The transversely oriented anterior conid in the p4 in *Chiyoumeryx* nov. gen. *shinaoensis* differs from the obliquely oriented one in *Chiyoumeryx* nov. gen. *flavimperatoris* nov. sp*.* In *Chiyoumeryx* nov. gen. *shinaoensis*, the posterolingual conid is vestigial on p4. *Chiyoumeryx* nov. gen. *shinaoensis* has no anterior cingulid, while in *Chiyoumeryx* nov. gen. *flavimperatoris* nov. sp. there is a tiny anterior cingulid. *Chiyoumeryx* nov. gen. *shinaoensis* possesses lower crowns than ?*Chiyoumeryx* nov. gen. *turgaicus*. *Chiyoumeryx* nov. gen. *flavimperatoris* nov. sp. possesses an ectostylid, which is absent in ?*Chiyoumeryx* nov. gen. *turgaicus*.

**Holotype.** IVPP V 6531, right mandible with p2-m3 and tooth socket of p1.

**Paratype.** IVPP V 6532, right fragmented maxillary with P4-M3.

**Additional material.** IVPP V 6533, right mandible with p2-m3 and tooth socket of i1-p1; IVPP V 6534, left fragments mandible with m1-m3; IVPP V 6535, right fragmented mandible with m1-m3; IVPP V 6536, left fragmented mandible with p4-m3; IVPP V 6537, right fragmented mandible with p4-m2; IVPP V 6538, left p4; IVPP V 6539, right maxillary with P3-M3; IVPP V 6540, right maxillary with P4-M2; IVPP V 6541, right maxillary with M2-M3; IVPP V 6542, left maxillary with P3-M1; IVPP V 6543, right maxillary with M1-M3; IVPP V 6544, Left M3; IVPP V 6545, left maxillary with P4-M3.

**Locality.** Shinao Basin, Panxian County, Southwestern Guizhou, China. Late Eocene.

**Description**

Mandible: The mandible is slender. The lower outline of the mandible is relatively straight. The thickness of the mandible is constant. The foramena mentale is located under the p1. The symphysis is long, starting between p1 and p2. The diastema between c and p1 is very short, smaller than p2 in length. The anterior part of the mandible is short, giving a massive aspect to the bone.

Lower tooth row: The root of the canine root is rounded while the incisor roots are laterally compressed. There is no diastema between c and i3. The p1 is mono-rooted. There is no diastema between p1 and p2. The premolars become more complex from p2 to p4. The p3 and p4 are of similar size. The premolars are narrow and very elongated. The anterior conid is weak on p2, oblique and of medium size on p3, and labio-lingually oriented and as wide as the tooth on p4. The mesolabial conid is as large as on p2 to only occupying the labial half of the p4. The anterolabial crisitid is located on the labial edge of the tooth. The mesolabial conid becomes a little bigger from p2 to p4 and moves from anterior to median. However, the posterior stylid remains short on all premolars, narrowing the posterior portion of the premolars. On p2, the posterolingual conid is marked and labio-lingually oriented, extending on the labial half of the posterior valley. On p3, it is bigger and reaches the lingual part of the tooth. On p4, it is almost absent. It does not join the posterolabial cristid. The posterior stylid is of similar size in all premolars reaching the lingual side of the premolars. The transverse cristid forms an oblique posterolingual ridge on p2. It is more developed on p3, forming a small wing very similar to the one observed on p4. Only the p4 bears a mesolingual conid. The transverse cristid does not directly fuse with the mesolingual conid, but joins a very short and posterolabially oriented cristid from the mesolingual conid. There is a big groove between the mesolingual conid and the transverse cristid. The conid is very anteriorly rounded. There is no anterior cingulid. The posterolabial cristid forms the labial edge of the tooth and continues with the posterolabial conid and the posterior stylid until reaching the lingual face.

The lower molars bear a small ectostylid and no posterior ectostylid on m3. The anterior cingulid is not very developed and seems to be of similar size as the posterior one. There is no premetacristid. The preprotocristid is very elongated, curved and stops on the lingual side of the molars leaving the trigonid lingually widely anteriorly open. There is no external postprotocristid. The internal postprotocristid is straight and short. It is oriented relatively labio-lingually on the m1 and a little posteriorly tilted on the m3. The prehypocristid almost fuses with the other cristids. There is an extremely small gap between the preentocristid and the prehypocristid. The internal postmetacristid is short and oblique. The internal postprotocristid fuses with the very short internal postmetacristid and preentocristid. The external postmetacristid is posteriorly oriented and gets smaller from m1 to m3. On unworn teeth, the metaconid bears an additional short “cristid” that is labio-lingually oriented (IVPP V 6531, IVPP V 6535). The entoconid is laterally compressed and bears a relatively elongated postentocristid, more marked on m1 and m2 than m3, on fresh teeth. All entocristids are straight. The posthypocristid is tilted a little backward and does not join the lingual face. The posterior opening of the posterior fossa is narrow. The posterior cingulid forms a posterior ridge on m1 that is nearly as high as the cristids. The posthypocristid stops along the distal part of the entoconid on m1 and m2 enclosing the posterior fossa. There is no distal cingulid on m3 third lobe. The hypoconulid forms the distal end of the m3. This cuspid is labio-lingually median. The prehypoconulidcristid is very rounded and fuses with the distal end of the posthypocristid. The posthypoconulidcristid is very short and straight. It ends at the median part of the back fossa of m3, leaving the fossa widely lingually open. This fossa is enlarged.

Upper tooth row: The P3 bears a very well-formed lingual cone: well-individualized, rounded, forming a lingual protrusion. The lingual and labial cones are relatively anteroposteriorly median. The posterior part of the P3 is pinched, while the anterior part is labiolingually widened due to the large lingual cone. The labial cone has a weakened labial rib and forms a concave labial wall. The posterolabial crista is a little concave. There is no anterolabial crista, the anterior style joining the labial cone. The anterior style is large occupying the labial half of the tooth. It possesses an anterolabial ridge forming a quite angular anterior portion of the tooth. Its posterior crista of the anterior style ends on the anterior part of the labial cone forming a straight portion of the labial wall. The curved anterior crista of the anterior style joins the anterolingual part of the labial cone. The posterolingual crista is relatively labio-lingually transverse and joins the posterior part of the labial cone. There is no cingulum. The posterior part of the P3 is relatively straight and lies along the anterior part of P4. There is no posterior style. The lingual and labial cones are median in P4. The labial cristae are straight. There is no labial rib on the labial cone. The labial wall is concave. There is no posterior style. There are very weak anterior and posterior cinguli. An additional posterior cingulum encloses the posterior part of the P4 joining the base of the distal posterolabial crista giving the P4 a triangular occlusal shape. This additional cingulum can be connected to the posterolingual cingulum. The posterolingual crista is not fused with the distal end of the posterolabial crista. The posterolingual crista is curved and goes on the posterior part of the labial cone, like on P3, enclosing the labial elements posteriorly. The intrusion of the P3 separates anteriolingual crista and anterior style. The anterior style is globular, but very low. The anterolabial crista connects the apex of the labial cone to the posterior part of the anterior style.

The upper molars are relatively square. The protocone becomes relatively bigger along the tooth row increasing the size of the molars from M1 to M3. The strong anterolingual cingulum surrounds the protocone in all molars. In IVPP V 6532, the lingual cingulum continues slightly to the metaconule. The entostyle is small on all molars. The lingual cusps, paracone and metacone, are not in line, especially for M2 and M3 on IVPP V 6545. The mesostyle becomes bigger and more globular from M1 to M3. The metacone is in the same axis than the paracone in M1 and becomes more oblique in M2 and M3, especially on IVPP V 6545. Thus on the M1 the postparacrista and the premetacrista reach the mesostyle on its lingual part, while only the premetacristia reaches the mesostyle on the other molars. On M2 and M3, the postparacrista terminates in the premetacrista more lingually. All cristae are straight. The paracristae are in line. The preparacrista reaches the parastyle on its posterior part. The paracone labial rib is little marked and there is no metacone labial rib. The parastyle is labio-lingually elongated and anteroposteriorly compressed. The parastyle becomes bigger from M1 to M3, and more labially extended. The preprotocrista is fused with the parastyle. The preparacrista and the premetaconulecrista are slightly anteriorly tilted. The postprotocrista is posteriorly oriented and short. On the M1, there is no fusion of the postprotocrista and the premetaconulecrista, leaving a groove between the two structures on fresh teeth, meeting basally. On M2, the postprotocrista reaches the premetaconulecrista and on M3 they fuse quite high. The fusion on M3 occurs centrally. The premetaconulecrista is elongated, stopping deep between the labial cusps, and is a little curved. The postmetaconulecrista is relatively straight and fuses with the distal part of the postmetacrista enclosing the posterior fossa. The metastyle is absent on the M1and M2 and big on the M3.

*Chiyoumeryx* nov. gen. *flavimperatoris* nov. sp.

Fig. S3

v 1961 cf. *Miomeryx* sp.—Xu: 316, 323, 324.

v pars 1982 *Lophiomeryx gracilis*—Miao: 532, tab. 3, fig. 9a-9b

v non 1982 ?*Lophiomeryx gracilis*—Miao: 536, fig.8.

*1983* *Lophiomeryx* sp.—Wang & Zhang: 122, 127.

v *1983* cf. *Miomeryx* sp.—Wang & Zhang: 123.

v *1997* *Miomeryx* sp.—Vislobokova: fig. 3

v pars *1997* *L. gracilis*—Vislobokova: fig. 3

v *1999* cf. *Miomeryx* sp.—Zhang, Long, Ji, & Ding: 7, tab. 5.

v pars *2000* *L. gracilis*—Guo, Dawson, and Beard: 247, tab. 2

v pars *2001* *L. gracilis*— Métais, Chaimanee, Jaeger, and Ducrocq: 239, 241

v *2007* *Miomeryx* sp.—Métais and Vislobokova: 194.

v pars *2012* *L. gracilis*—Mennecart: 62

**ZooBank LSID.** urn:lsid:zoobank.org:act:1DF6F58C-F08B-4657-BD4A-7C597653926F

**Etymology.** meaning yellow (*flavor-*) emperor (*imperatoris*) in latin. Chiyou fought with the Yellow Emperor, the ancestor of Chinese, but was defeated.

**Diagnosis.** *Chiyoumeryx* nov. gen. *flavimperatoris* nov. sp. shows the above-mentioned characteristics of the genus. *Chiyoumeryx* nov. gen. *flavimperatoris* nov. sp. is smaller than *Chiyoumeryx* nov. gen. *shinaoensis* and ?*Chiyoumeryx* nov. gen. *turgaicus*. The p4 of *Chiyoumeryx* nov. gen. *flavimperatoris* nov. sp. differs from *Chiyoumeryx* nov. gen. *shinaoensis* by an oblique anterior conid, which is labio-lingually oriented in the larger species. A very short posterolingual conid is located between the posterolabial cristid and the transverse cristid in the p4 of *Chiyoumeryx* nov. gen. *flavimperatoris* nov. sp., while it is absent on *Chiyoumeryx* nov. gen*.* *shinaoensis*. In *Chiyoumeryx* nov. gen. *flavimperatoris* nov. sp., there is a tiny anterior cingulid, while it is absent in *Chiyoumeryx* nov. gen. *shinaoensis*.

**Holotype.** IVPP V 6547, right mandible with p4-m3 (previously attributed to *Lophiomeryx gracilis* in Miao 1982).

**Paratype.** IVPP V 6548, left mandible with p4-m3 (previously attributed to *Lophiomeryx gracilis* in Miao 1982).

**Additional material.** IVPP V 2600, left p4-m2 (previously attributed to cf. *Miomeryx* sp. in Xu 1961).

**Localities.** Yangjiachong locality lying in the Caijiachong marls, Qujing, Yunnan, China; Shinao Basin, Panxian County, Southwestern Guizhou, China. Late Eocene.

**Description**

*Chiyoumeryx* nov. gen. *flavimperatoris* nov. sp. is a small ruminant. The mandible is slender. It is a little doso-ventrally concave like the tooth row. The incisura vasorum is shallow and enlarged.

The p4 is laterally compressed. The anterior conid is oblique. There is a tiny anterior cingulid. The mesolabial conid is high and central. The anterolabial crisitid is located on the labial edge of the tooth and joins the posterior part of the anterior conid. The transverse cristid is oblique and posteriorly tilted. It does not directly fuse with the mesolingual conid, but joins a very short posterolabialy oriented cristid from the mesolingual conid. There is a big groove between the mesolingual conid and the transverse cristid. There is no additional cristid on the mesolingual conid, which is a well-rounded conid. The posterolabial cristid meets the posterior stylid, which ends on the lingual side of the teeth. The posterolingual conid is an isolated structure located median between the posterolabial cristid and the transverse cristid. It size may vary from a small, located at the base of the mesolingual conid, to big, reaching the lingual part of the tooth.

The lower molars lack a premetacristid. The metaconid is posterior in the trigonid and stops at the level of the labio-lingually oriented internal postprotocristid. The ectostylid is very small on all molars. There is no external postprotocristid. The preprotocristid stops in the axis of the metaconid, accentuating the lingual bulky aspect of the lingual cuspids. The trigonid is broadly open to anterior. The small anterior cingulid gets bigger from m1 to m3. The posterior one is big in m1 and m2, but absent in m3. The entoconid is laterally compressed. The metaconid's anterior part is well-rounded. There are two postmetacristids. The external postmetacristid is elongated forming a deep groove between the internal and external postmetacristid. The internal one is relatively labialy oriented and joins the internal postprotocristid forming a triple point with the fusion of the straight and elongated preentocristid. The postentocristid is very short and does not fuse with the posthypocristid leaving the posterior basin open. The long prehypocristid ends in the medial part of the postprotocristid. The trigonid is less broad than the talonid on m1, but of similar size on m2 and m3. The hypoconulid forms the distal part of the tooth, relatively medially located on m3. The back fossa of m3 is a little oblique, narrow, and slightly open anterolingually. On m3, the posthypocristid does not reach the lingual part of the tooth, but the distal part is directly connected with the prehypoconulidcristid. Both pre- and posthypoconulidcristids are relatively straight and parallel forming a narrow and elongated back fossa of m3.

Family Tragulidae Milne-Edwards, 1864

Genus *Iberomeryx* Gabunia, 1964

**Diagnosis (modified from Mennecart et al. 2011).** Small-sized ruminant with upper molars possessing the following combination of characters: well-marked parastyle and mesostyle in small-column shape; strong paracone rib; metacone rib absent; metastyle absent; unaligned external walls of metacone and paracone; strong postprotocrista stopping against the anterior side of the premetaconulecrista; continuous lingual cingulum, stronger under the protocone. Lower dental formula is primitive (3–1–4–3) with non-molarized premolars. Tooth c is adjacent to i3. Tooth p1 is single-rooted, reduced and separated from c and p2 by a short diastema. The premolars have a well-developed anterior conid. Teeth p2–p3 display a distally bifurcated mesolabial conid. Tooth p3 is the largest premolar. Tooth p4 displays no mesolingual conid and a large posterior valley. Regarding the lower molars, the trigonid and talonid are lingually open with a trigonid more tapered than the talonid. The anterior fossa is open, due to a forward orientation of the preprotocristid and the presence of a paraconid. The internal postprotocristid is oblique and the external postprotocristid reaches the prehypocristid. The internal postprotocristid, postmetacristid and preentocristid are fused and Y-shaped. Protoconid and metaconid display a weak *Tragulus* fold and a well-developed *Dorcatherium* fold, respectively. The mandible displays a regularly concave ventral profile in lateral view, a marked incisura vasorum, a strong mandibular angular process, a vertical ramus, and a stout condylar process.

**Type species.** *Iberomeryx parvus* Gabunia, 1964 from Benara (Georgia), Late Oligocene (Lucas & Emery, 1999)

**Included species.** *I. minor* (Filhol, 1882)

*Iberomeryx* *miaoi* nov. sp.

Fig. S4

v pars 1982 ?*Lophiomeryx gracilis* —Miao: 536, fig.8.

**ZooBank LSID.** urn:lsid:zoobank.org:act:EE3F88E9-0EAF-4EC6-A46F-8623241E614B

**Diagnosis.** *Iberomeryx* with a very large paraconid, which is smaller in *Iberomeryx minor* and *Iberomeryx parvus*. The metastylid is not strong but is more developed than in the other species. The ectostylid is big on m1, smaller on m2 and absent on m3, while *I. minor* displays an ectostylid on all molars and *I. parvus* none at all. *Iberomeryx* *miaoi* nov. sp. is of similar size to *I. minor* and its m2 is smaller than the one of *I. parvus*. It differs from *I. minor* by a thin anterior cingulid. Moreover, its protoconid is positioned slightly more anterior than in *I. parvus*. The molars appear to be more massive and bulkier in this species than in *I. minor* and *I. parvus.s*

**Holotype.** IVPP V 6551, left mandible with m1-m3 (only specimen known). m1 5.1x3.5, m2 5.2x4.1, m3 8.0x4.0

**Etymology.** We dedicate this species to Prof. Miao Desui who was the first to describe the Shinao fauna.

**Locality and horizon.** Shinao Basin, Panxian County, Southwestern Guizhou, China. Late Eocene.

**Description**

The molars are bunodont with very rounded and bulky cuspids. m1 and m2 are of similar length, but m2 is wider than m1. The m1 is not well preserved. Thus no detailed description can be given. Nevertheless, m1 and m2 display the same morphological pattern. The trigonid is anteriorly open since there is no premetacristid. There is a prominent paraconid on the anterolabial part of the teeth. It gets smaller from m1 to m3. The paraconid continues to the lingual edge of each teeth forming a cristid that can be described as a preparacristid. The paraconid forms the anterior-most part of each teeth, which are anteriorly well rounded. The internal and external postmetacristids are well developed. The anterior cingulid is very small; the posterior one is very strong. The external postmetacristid is posteriorly oriented and the internal postmetacristid is a little backwardly tilted. Both the external and internal postmetacristids are very short. The preprotocristids are longer than the postmetacristids. The preprotocristid is anteriorly oriented and fuses with the paraconid. The postprotocristid is splitted in internal and external branches. The internal postprotocristid is a little backward tilted. It becomes longer from m1 to m3. The internal postprotocristid and the internal postmetacristid meet in the median part of the tooth, forming a cristid a little lingually tilted and joining the preentocristid at the base of the entoconid. The preentocristid is very low on the globular entoconid and anteriorly oriented. The postentocristid is very short on m1 and m2 and even absent on m3 leaving the posterior basin open on the lower molars. There are two postprotocristid. The external postprotocristid, only observed on m2, is very shallow and almost parallel to the internal postprotocristid. Both hypocristids are of similar size and the longest cristids on the lower molars. On m2, the prehypocristid is oblique and fuses with the external postprotocristid forming a *Tragulus* fold (sensu Sánchez et al. 2014). The posthypocristid is transverse on m1 to backward tilted on m2 and m3. On m1 and m2, the posthypocristid ends on the lingual side of the tooth and do not fuse with the short postentocristid. The lower molars lack an entostylid. On m3, the posthypocristid stops before joining the lingual part of the tooth. The posthypocristid and the large posterior cingulid fuse together on the distolingual end. The ectostylid is big on m1 to absent on m3. There is no posterior ectostylid on the m3. The back fossa of m3 is anterolingually open. The hypoconulid forms the posteriorlingual edge of the tooth. The back fossa of m3 is a little oblique. The prehypoconulidcristid is straight and fuses with the posterior part of the posthypocristid. The posthypoconulidcristid is longer and curved stopping at the base of the distal end of the posthypocristid. The back fossa is relatively large and forms a buckle almost closed.

**Bibliography**

H. Filhol, Découverte de quelques nouveaux genres de mammifères fossiles dans les dépôts de phosphate de chaux de Quercy. *Comptes Rendus de l’Académie des Sciences* **94**, 138–139 (1882).

K. K. Flerow, On the remains of the Ungulata from Betpakdala. *Comptes Rendus de l’Académie des Sciences* **21**, 95–96 (1938).

W. H. Flower, On the arrangement of the orders and families of existing Mammalia. *Proceedings of the Zoological Society of London* **39**, 178–186 (1883).

L. Gabunia, The Oligocene mammalian fauna of Benara. Academy of Sciences of URSS, (1964) pp. 268.

J. Guo, M. Dawson, K. C. Beard, *Zhailimeryx*, a new Lophiomerycid Artiodactyl (Mammalia) from the Late Middle Eocene of Central China and early evolution of ruminants. *Journal of Mammalian Evolution* **7**, 239–258 (2000).

C. M. Janis, Grades and Clades in Hornless Ruminants Evolution: The Reality of the Gelocidae and the Systematic Position of *Lophiomeryx* and *Bachitherium*. *Journal of* *Vertebrate Paleontology* **7**, 200–216 (1987).

C. von Linnaeus, Systema naturæ per regna tria naturæ, secundum classes, ordines, genera, species, cum characteribus, differentiis, synonymis, locis. *Editio decima reformat*. 1758.

S. G. Lucas, R. J. Emery, Taxonomy and biochronological significance of *Paraentelodon*, a giant *Entelodon* (Mammalia, Artiodactyle) from the Late Oligocene of Eurasia. *Journal of Vertebrate Paleontology* **19**, 160–168 (1999)

B. Mennecart, The Ruminantia (Mammalia, Cetartiodactyla) from the Oligocene to the Early Miocene of Western Europe: systematics, palaeoecology and palaeobiogeography. *GeoFocus* **32**, 1–263 (2012).

G. Métais, I. Vislobokova, “Basal ruminants” in *The evolution of artiodactyls*, D. R. Prothero, S. C. Foss, Eds. (The Johns Hopkins University Press, 2007), pp. 189–212.

G. Métais, Y. Chaimanee, J.-J. Jaeger, S. Ducrocq, New remains of primitive ruminants from Thailand: evidence of the early evolution of the Ruminantia in Asia. *Zoologica Scripta* **30**, 231–248 (2001).

D. Miao, Early Tertiary fossil mammals from the Shinao Basin, Panxian County, Guizhou Province. *Acta Palaeontol. Sinica* **21**, 526–536 (1982).

A. Milne-Edwards, Recherches anatomiques, zoologiques et paléontologiques sur la famille des chevrotains. *Annales de Sciences Naturelles de Paris* **5**, 1–167 (1864).

R. Owen, Description f teeth and proportion of jaws of two extinct Anthracotherioid quadrupeds (*Hyopotamis vectianus* and *Hyopotamus bovinus*) discovered by the Marchioness of Hastings in the Eocene deposits on the N.W. coast of the Isle of Wight: with an attempt to develop Cuvier’s idea of the classificiation of the pachyderms by the number of their toes. *Quaterly Journal of the Geological Society of London* **4**, 103–141 (1848).

G. A. Scopoli, “Introductio ad Historiam Naturalem Sistens Genera Lapidum, Plantarum, et Animalium: Hactenus Detecta, Caracteribus Essentialibus Donata” in *Tribus Divisa, Subinde ad Leges Naturae*, G. A. Scopoli, W. Gerle, Eds. (Apud Wolfgangum Gerle, 1777), pp. 1–540.

I. A. Vislobokova, *Eocene-early miocene ruminants in Asia*, J.-P. Aguilar, S. Legendre, J. Michaux, Eds. (BiochroM, 1997), pp. 215–223.

B. Wang, Y. Zhang, New finds of fossils from Paleogene of Qujing, Yunnan. *Vertebrata PalAsiatica* **21**, 119–128 (1983).

Y.-X. Xu, Some Oligocene mammals from Chuching, Yunnan. *Vertebrata PalAsiatica* **4**, 315–325 (1961).

Y. Zhang, Y. Long, H. Ji, S. Ding, The Cenozoic deposits of the Yunnan region. *Professional Papers in Stratigraphy and Paleontology* **7**, 1–21 (1999).

**Figures & captions**


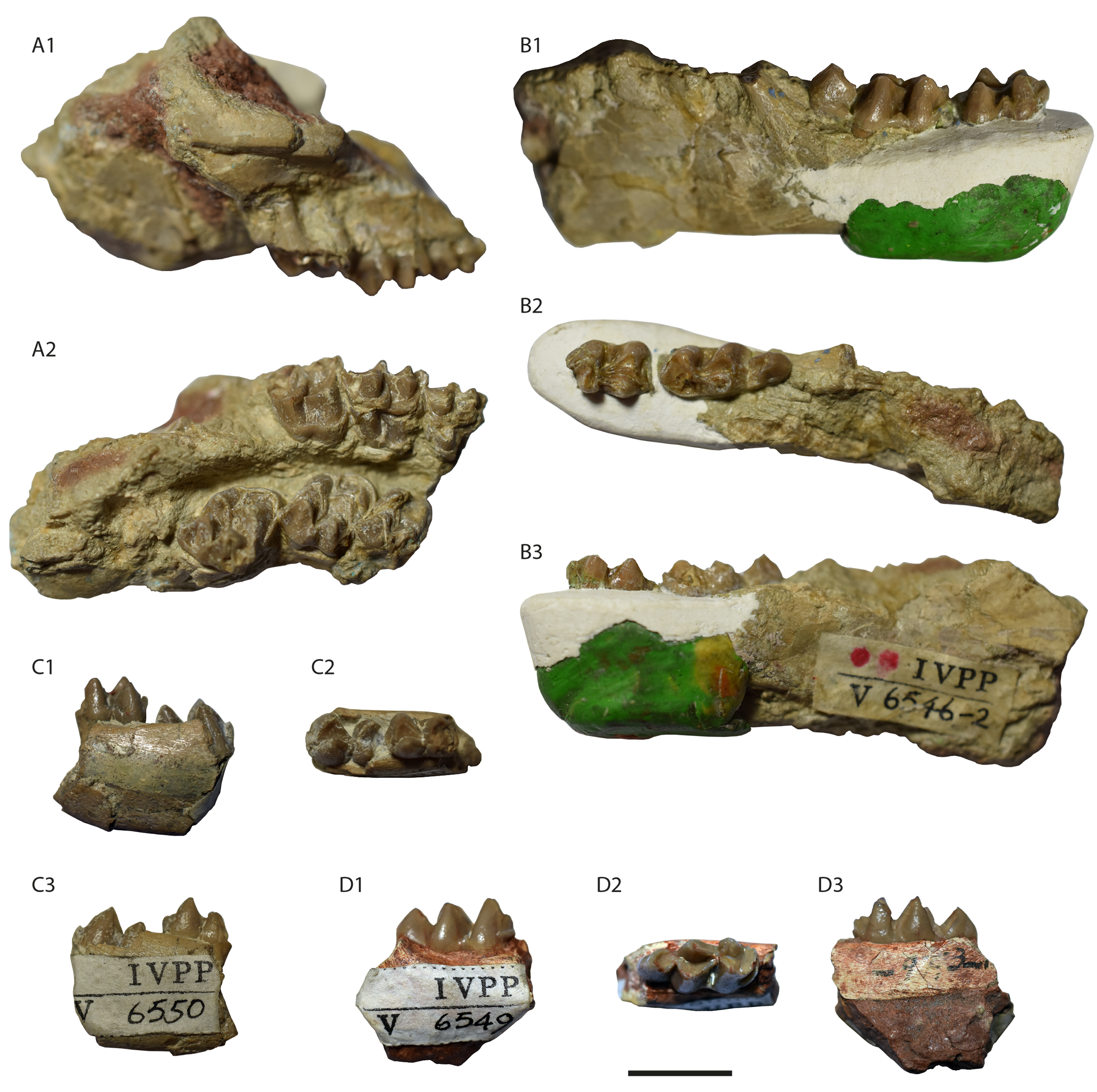


**Fig. S1.** *Krabimeryx gracilis* nov. comb. (Miao, 1982). **A** IVPP V 6546-1 (holotype), partial skull with right and left M1-M3; **B** IVPP V 6546-2 (holotype), right fragmented mandible with m2-m3; **C** IVPP V 6550 left fragmented mandible with m1-m2; **D** IVPP V 6549, right m3 on fragmented mandible; **E** IVPP V 26638, right m1; in **1** labial, **2** occlusal, and **3** lingual views. Scale bare is 1cm.


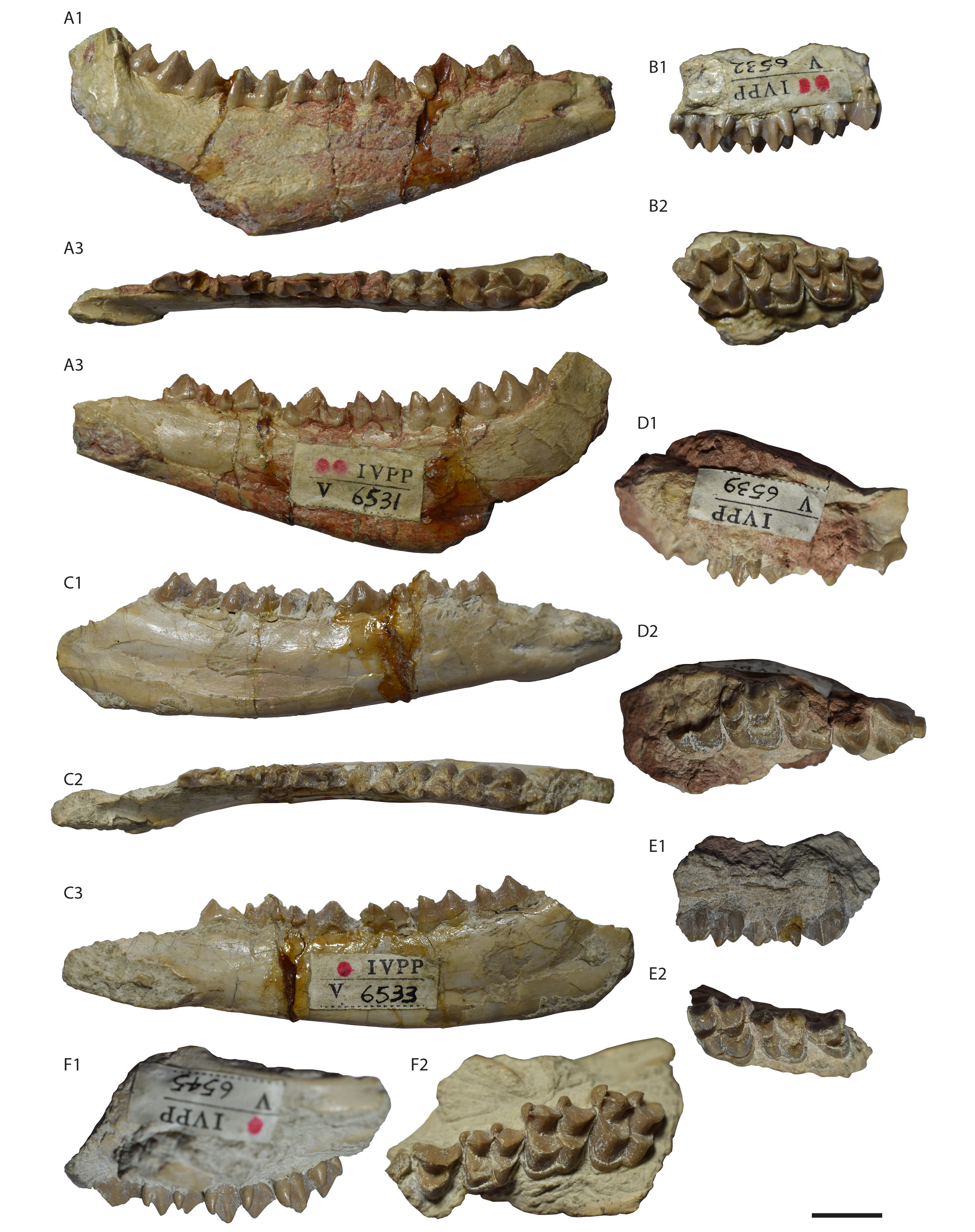


**Fig. S2.** *Chiyoumeryx* nov. gen. *shinaoensis* (Miao, 1982). **A** IVPP V 6531 (holotype), right mandible with p2-m3 and tooth socket of p1; **B** IVPP V 6532 (paratype), right fragmented maxillary with P4-M3; **C** IVPP V 6533, right mandible with p2-m3 and tooth socket of i1-p1; **D** IVPP V 6539, right maxillary with P3-M3; **E** IVPP V 6540, right maxillary with P4-M2; **F** IVPP V 6545, left maxillary with P4-M3; in **1** labial, **2** occlusal, and **3** lingual views. Scale bare is 1cm.


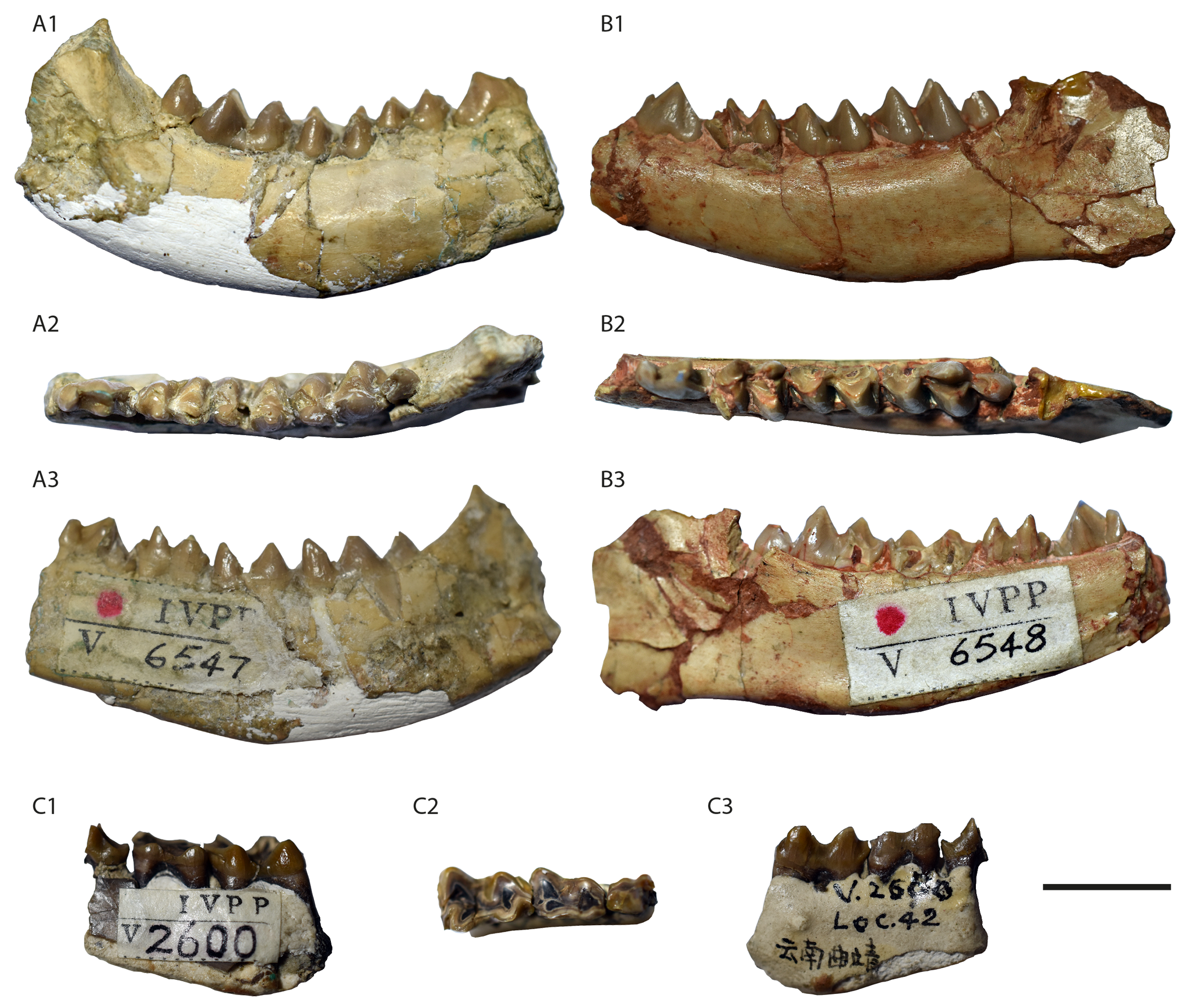


**Fig. S3.** *Chiyoumeryx* nov. gen. *flavimperatoris* nov. sp. **A** IVPP V 6547 (holotype), right mandible with p4-m3; **B** IVPP V 6548 (paratype), left mandible with p4-m3; **C** IVPP V 2600, left p4-m2; in **1** labial, **2** occlusal, and **3** lingual views. Scale bare is 1cm.


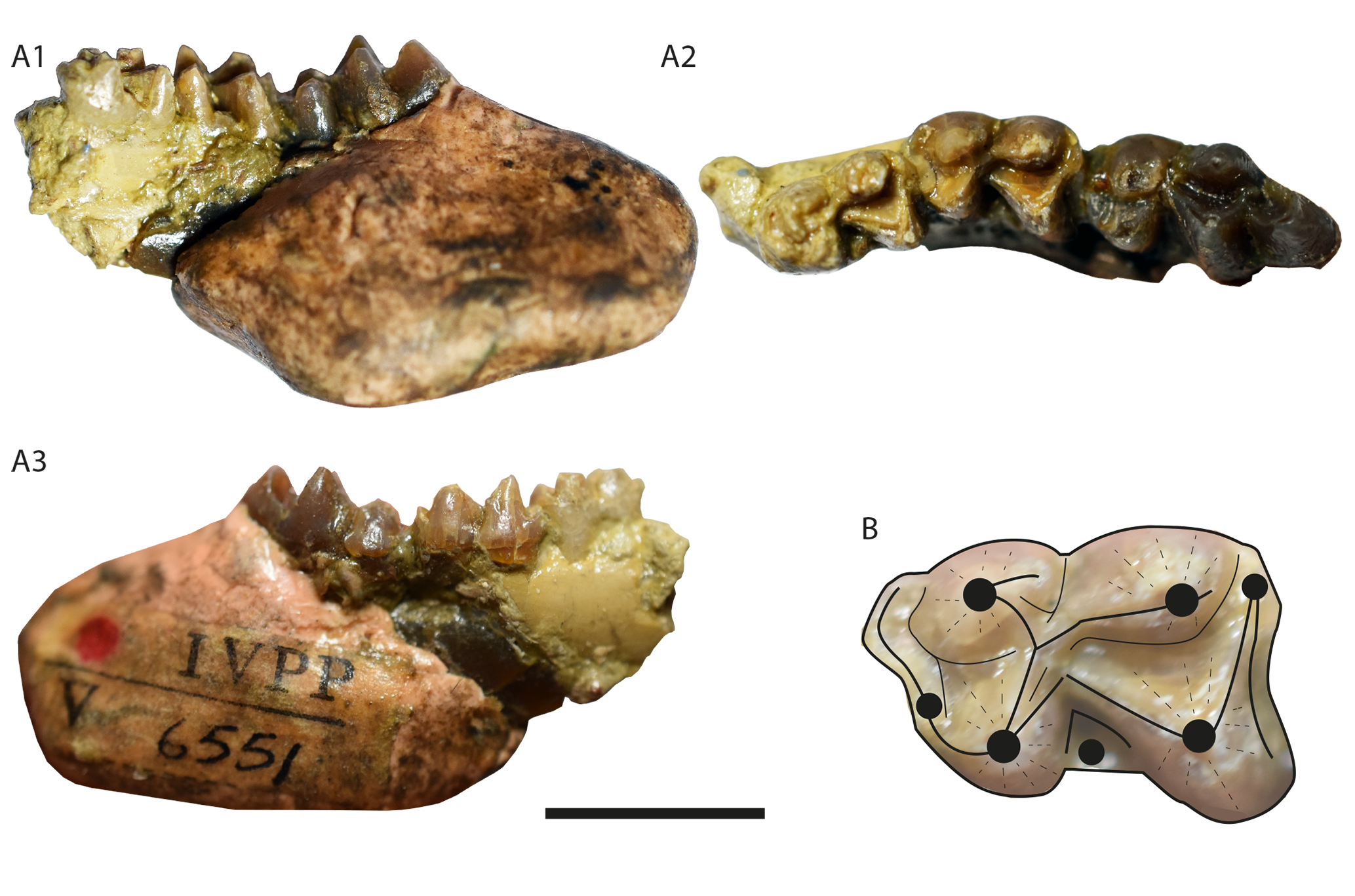


**Fig. S4.** *Iberomeryx* *miaoi* nov. sp. **A** IVPP V 6551 (holotype), left mandible with m1-m3; in **1** labial, **2** occlusal, and **3** lingual views. Scale bare is 1cm. **B** interpretative drawing of the molar structures.
