# Supplementary figures and images for: Ruminants reveal Eocene Asiatic palaeobiogeographical provinces as the origin of diachronous mammalian Oligocene dispersals into Europe
Source: Sci Rep. 2021 Sep 6;11:17710. doi: 10.1038/s41598-021-96221-x (PMC8421421; doi:10.1038/s41598-021-96221-x)

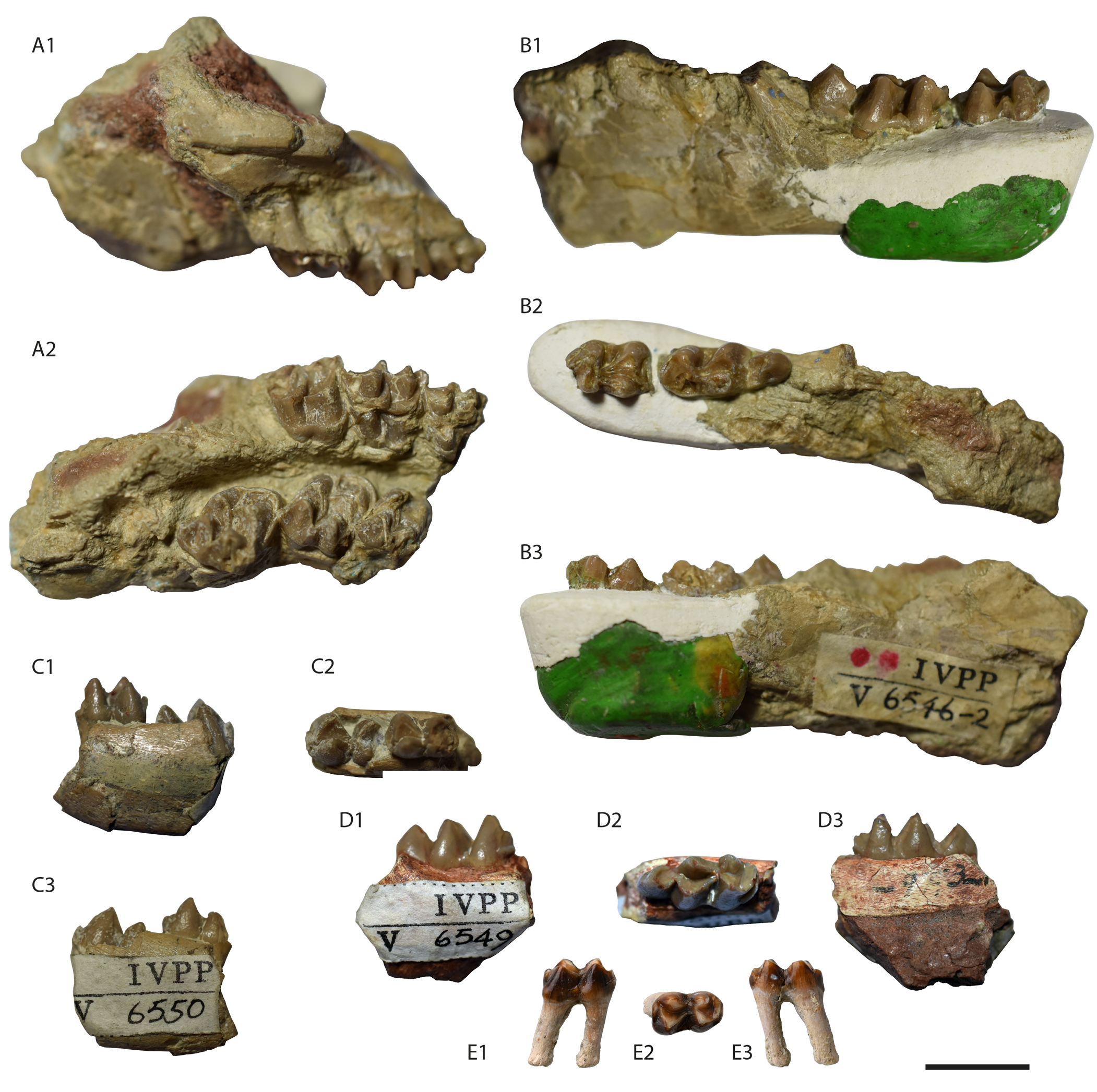

Supplement: Supplementary file 1 — Supplementary Information. [file 41598_2021_96221_MOESM1_ESM.zip › Supplementary data 1/Figure S1.tif]

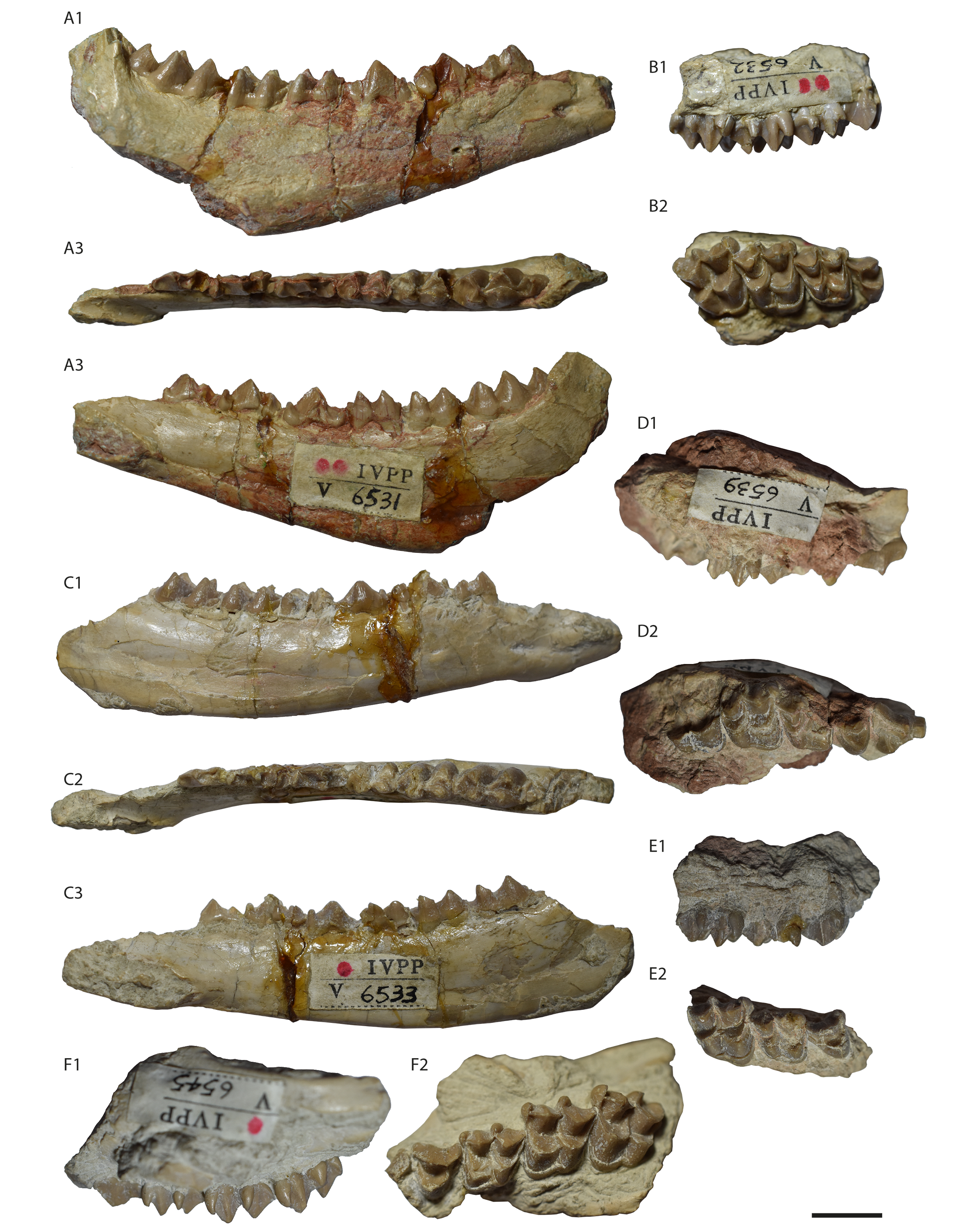

Supplement: Supplementary file 1 — Supplementary Information. [file 41598_2021_96221_MOESM1_ESM.zip › Supplementary data 1/Figure S2.tif]

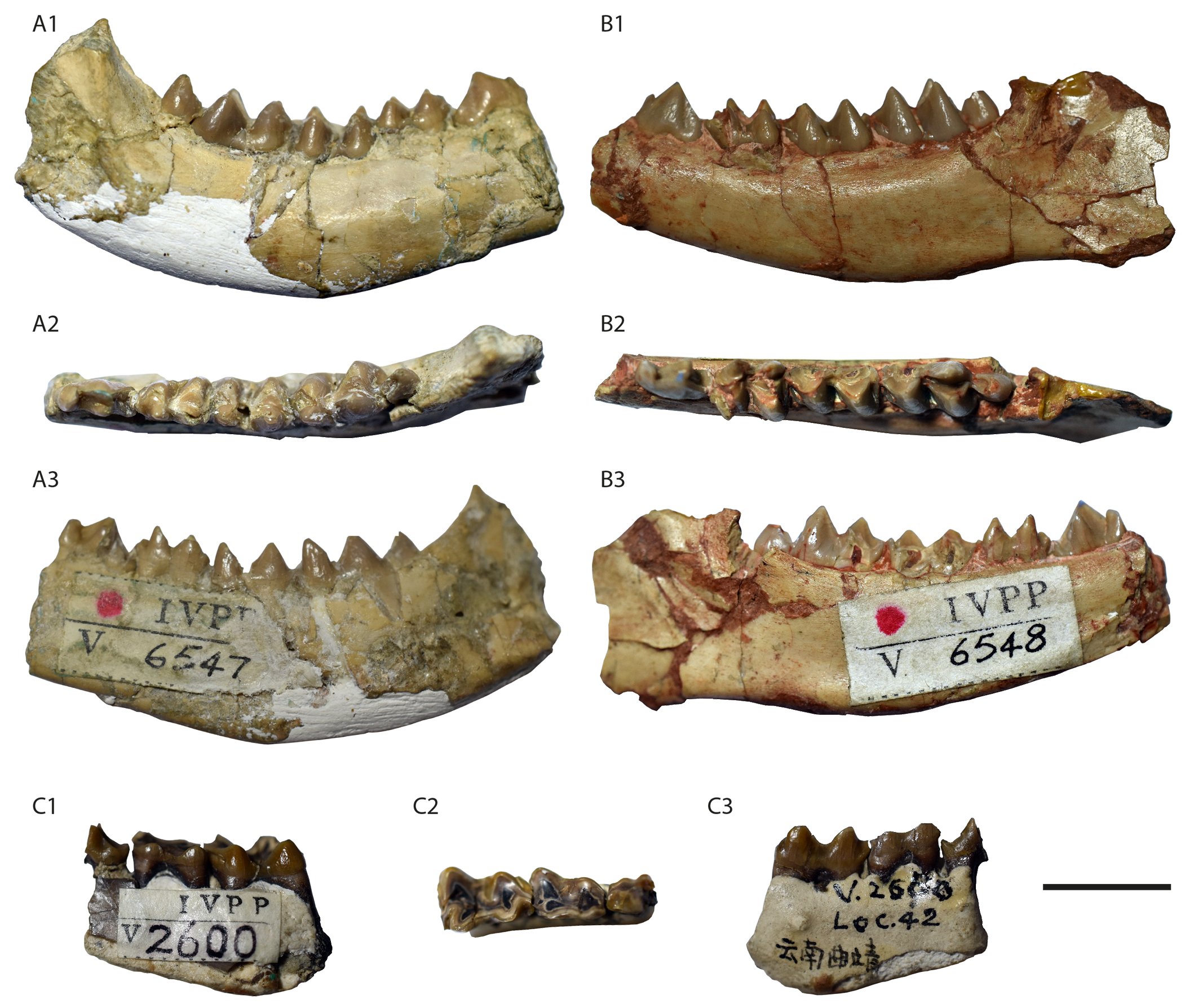

Supplement: Supplementary file 1 — Supplementary Information. [file 41598_2021_96221_MOESM1_ESM.zip › Supplementary data 1/Figure S3.tif]

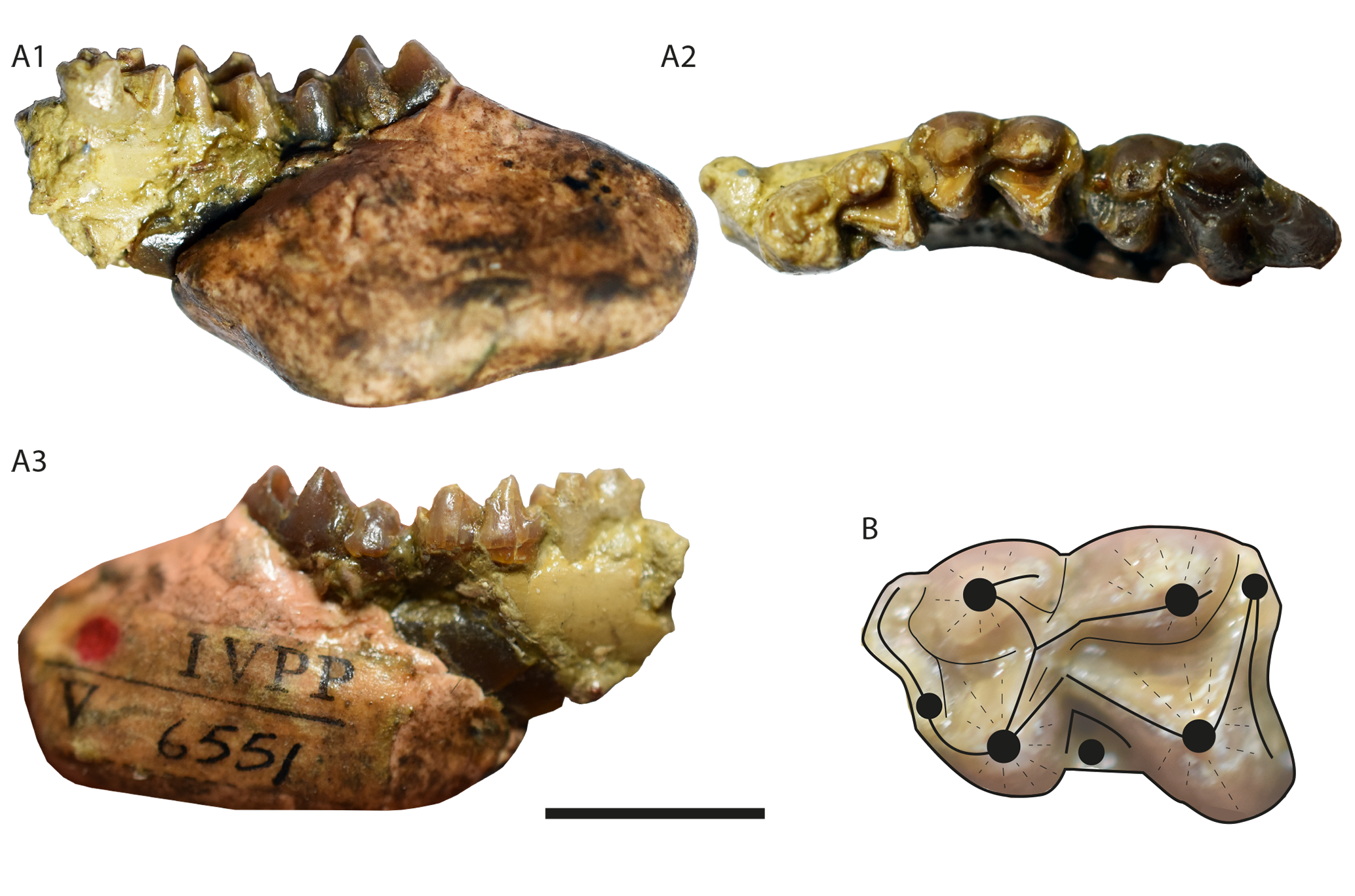

Supplement: Supplementary file 1 — Supplementary Information. [file 41598_2021_96221_MOESM1_ESM.zip › Supplementary data 1/Figure S4.tif]
